# Transcriptomic profiles of multiple organ dysfunction syndrome phenotypes in pediatric critical influenza

**DOI:** 10.3389/fimmu.2023.1220028

**Published:** 2023-07-18

**Authors:** Tanya Novak, Jeremy Chase Crawford, Georg Hahn, Mark W. Hall, Simone A. Thair, Margaret M. Newhams, Janet Chou, Peter M. Mourani, Keiko M. Tarquinio, Barry Markovitz, Laura L. Loftis, Scott L. Weiss, Renee Higgerson, Adam J. Schwarz, Neethi P. Pinto, Neal J. Thomas, Rainer G. Gedeit, Ronald C. Sanders, Sidharth Mahapatra, Bria M. Coates, Natalie Z. Cvijanovich, Kate G. Ackerman, David W. Tellez, Patrick McQuillen, Stephen C. Kurachek, Steven L. Shein, Christoph Lange, Paul G. Thomas, Adrienne G. Randolph

**Affiliations:** ^1^ Department of Anesthesiology, Critical Care and Pain Medicine, Boston Children’s Hospital, Boston, MA, United States; ^2^ Department of Anaesthesia, Harvard Medical School, Boston, MA, United States; ^3^ National Institute of Allergy and Infectious Diseases (NIAID), Centers of Excellence for Influenza Research and Response (CEIRR), Center for Influenza Disease and Emergence Response (CIDER), Athens, GA, United States; ^4^ National Institute of Allergy and Infectious Diseases (NIAID), Centers of Excellence for Influenza Research and Response (CEIRR), St. Jude Children's Research Hospital, Memphis, TN, United States; ^5^ Department of Immunology, St Jude Children’s Research Hospital, Memphis, TN, United States; ^6^ Channing Division of Network Medicine, Department of Medicine, Brigham and Women’s Hospital, Boston, MA, United States; ^7^ Division of Critical Care Medicine, Department of Pediatrics, Nationwide Children’s Hospital, Columbus, OH, United States; ^8^ Division of Biomedical Informatics Research, Department of Medicine, Stanford University School of Medicine, Stanford, CA, United States; ^9^ Division of Immunology, Boston Children’s Hospital, Harvard Medical School, Boston, MA, United States; ^10^ Department of Pediatrics, Harvard Medical School, Boston, MA, United States; ^11^ Department of Pediatrics, Section of Critical Care Medicine, University of Arkansas for Medical Sciences and Arkansas Children’s Research Institute, Little Rock, AR, United States; ^12^ Division of Critical Care Medicine, Department of Pediatrics, Emory University School of Medicine, Children’s Healthcare of Atlanta, Atlanta, GA, United States; ^13^ Department of Anesthesiology Critical Care Medicine, Children’s Hospital Los Angeles, Los Angeles, CA, United States; ^14^ Division of Critical Care Medicine, Department of Pediatrics, Baylor College of Medicine, Houston, TX, United States; ^15^ Nemours Children’s Hospital Delaware, Critical Care Medicine, Wilmington, DE, United States; ^16^ Pediatric Critical Care Medicine, St. David’s Children’s Hospital, Austin, TX, United States; ^17^ Department of Pediatrics, Children’s Hospital of Orange County, Orange, CA, United States; ^18^ Department of Anesthesiology and Critical Care Medicine, Children’s Hospital of Philadelphia, Philadelphia, PA, United States; ^19^ Department of Pediatrics, Penn State Health Children’s Hospital, Penn State University College of Medicine, Hershey, PA, United States; ^20^ Pediatric Critical Care, Milwaukee Hospital-Children’s Wisconsin, Milwaukee, WI, United States; ^21^ Section of Pediatric Critical Care, Department of Pediatrics, University of Arkansas for Medical Sciences and Arkansas Children’s Research Institute, Little Rock, AR, United States; ^22^ Pediatric Critical Care Medicine, Children’s Hospital & Medical Center Omaha, University of Nebraska Medical Center, Omaha, NE, United States; ^23^ Division of Critical Care Medicine, Department of Pediatrics, Northwestern University Feinberg School of Medicine, Ann & Robert H. Lurie Children’s Hospital of Chicago, Chicago, IL, United States; ^24^ Division of Critical Care Medicine, UCSF Benioff Children’s Hospital, Oakland, CA, United States; ^25^ Department of Pediatrics, University of Rochester/UR Medicine Golisano Children’s Hospital, Rochester, NY, United States; ^26^ Pediatric Critical Care Medicine, Phoenix Children’s Hospital, Phoenix, AZ, United States; ^27^ Department of Pediatrics, Benioff Children’s Hospital, University of California, San Francisco, San Francisco, CA, United States; ^28^ Department of Critical Care, Children’s Specialty Center, Children’s Minnesota, Minneapolis, MN, United States; ^29^ Division of Pediatric Critical Care Medicine, University Hospitals Rainbow Babies and Children’s Hospital, Cleveland, OH, United States; ^30^ Department of Biostatistics, T.H. Chan School of Public Health, Harvard University, Boston, MA, United States

**Keywords:** influenza, sepsis, organ failure, pediatric intensive care, neutrophil degranulation, MODS, neutrophil transcripts, critical care

## Abstract

**Background:**

Influenza virus is responsible for a large global burden of disease, especially in children. Multiple Organ Dysfunction Syndrome (MODS) is a life-threatening and fatal complication of severe influenza infection.

**Methods:**

We measured RNA expression of 469 biologically plausible candidate genes in children admitted to North American pediatric intensive care units with severe influenza virus infection with and without MODS. Whole blood samples from 191 influenza-infected children (median age 6.4 years, IQR: 2.2, 11) were collected a median of 27 hours following admission; for 45 children a second blood sample was collected approximately seven days later. Extracted RNA was hybridized to NanoString mRNA probes, counts normalized, and analyzed using linear models controlling for age and bacterial co-infections (FDR q<0.05).

**Results:**

Comparing pediatric samples collected near admission, children with Prolonged MODS for ≥7 days (n=38; 9 deaths) had significant upregulation of nine mRNA transcripts associated with neutrophil degranulation (*RETN, TCN1, OLFM4, MMP8, LCN2, BPI, LTF, S100A12, GUSB)* compared to those who recovered more rapidly from MODS (n=27). These neutrophil transcripts present in early samples predicted Prolonged MODS or death when compared to patients who recovered, however in paired longitudinal samples, they were not differentially expressed over time. Instead, five genes involved in protein metabolism and/or adaptive immunity signaling pathways (*RPL3, MRPL3, HLA-DMB, EEF1G*, *CD8A*) were associated with MODS recovery within a week.

**Conclusion:**

Thus, early increased expression of neutrophil degranulation genes indicated worse clinical outcomes in children with influenza infection, consistent with reports in adult cohorts with influenza, sepsis, and acute respiratory distress syndrome.

## Introduction

Severe lower respiratory tract infections (LRTI) are a leading cause of hospitalization and preventable death in children worldwide (1). In the US alone, from 2010-2020, an average of one in 4,000 children under 18 years old were hospitalized with severe influenza virus infection and 1,327 influenza-related deaths were reported (2, 3). Multiple organ dysfunction syndrome (MODS) is an uncommon but life-threatening and sometimes fatal complication of severe influenza infection in children (4). MODS is a heterogeneous syndrome with established clinical criteria but poorly defined pathophysiology. Therefore, a better understanding of the immunobiology of influenza-related pediatric MODS, including its development, persistence, and recovery, could lead to improved therapeutic interventions and prognostic stratification.

In this multicenter cohort of children with influenza-related critical illness, we previously reported that patients with MODS had a higher frequency of immunosuppression, as measured by reduced responsiveness to *ex-vivo* stimulation of whole blood using toll-like receptor 4 (TLR4) and retinoic acid-inducible gene I (RIG-I) pathway agonists compared to those who did not develop MODS (5, 6). They also, somewhat paradoxically, had evidence of greater systemic inflammation across multiple biomarkers (7). In this same cohort, we now evaluate whole blood messenger RNA (mRNA) gene expression profiles of a curated list of genes involved in immune response, inflammation, and metabolism among other functions using NanoString. We sought to identify genes predicting patients with Prolonged MODS and/or death compared to those that recovered from or never developed MODS. We also used available longitudinal data to provide insight into differences in gene expression over time associated with recovery from organ dysfunction.

## Materials and methods

Biological samples for this study came from children previously enrolled in the Pediatric Intensive Care Influenza (PICFLU) Genetic Epidemiology and Immune Response Study (https://picflu.org) from sites across the Pediatric Acute Lung Injury and Sepsis Investigators (PALISI) network (www.palisi.org). The study was approved by the Institutional Review Boards at each hospital site, and informed consent was obtained from at least one parent or guardian prior to participation. The PICFLU patients included in this investigation had laboratory confirmed community-acquired influenza lower respiratory tract infection and had been admitted to one of the 30 different Pediatric Intensive Care Units (PICU) between March 2010 and March 2017. Inclusion criteria required support with invasive or non-invasive mechanical ventilation and/or vasoactive support. To minimize the confounding effects of comorbidities on influenza outcomes, patients with pre-existing immune suppressive conditions and other chronic cardiorespiratory and metabolic illnesses were excluded (see **Supplementary Material** for inclusion/exclusion criteria).

Blood (≤ 2.5 mL) from consented patients was collected in PAXgene® tubes (BD Diagnostics, PreAnalytical Systems, Franklin Lakes, NJ) as soon as possible after PICU admission. When possible, a second blood sample was collected between 6 to 8 days after the first. Samples were kept at room temperature (up to 24 hours, minimum of 2 hours) then frozen at -80°C. Sites shipped samples on dry ice to Boston Children’s Hospital for analysis. The **Supplementary Material** details the RNA extraction procedures.

### NanoString custom gene panel

A custom NanoString gene panel was designed for 469 mRNA targets incorporating genes known for moderating inflammation, cytokines, purinergic signaling, or those specifically shown to be associated with acute respiratory distress syndrome (ARDS), sepsis, or influenza from previous publications (See **Supplemental Material**, **Table S1** Gene Source). Seven housekeeping (HK) genes were included based on similar studies (8–10). Samples were prepared using Reporter and Capture ProbeSets (see **Supplemental Methods**) for details then loaded individually onto the NanoString cartridge. Non-amplified mRNA was quantified using the nCounter® SPRINT Profiler (NanoString Technologies, Inc., Seattle, WA) in batches of 12.

### Organ dysfunction assessment

**Figure 1:**
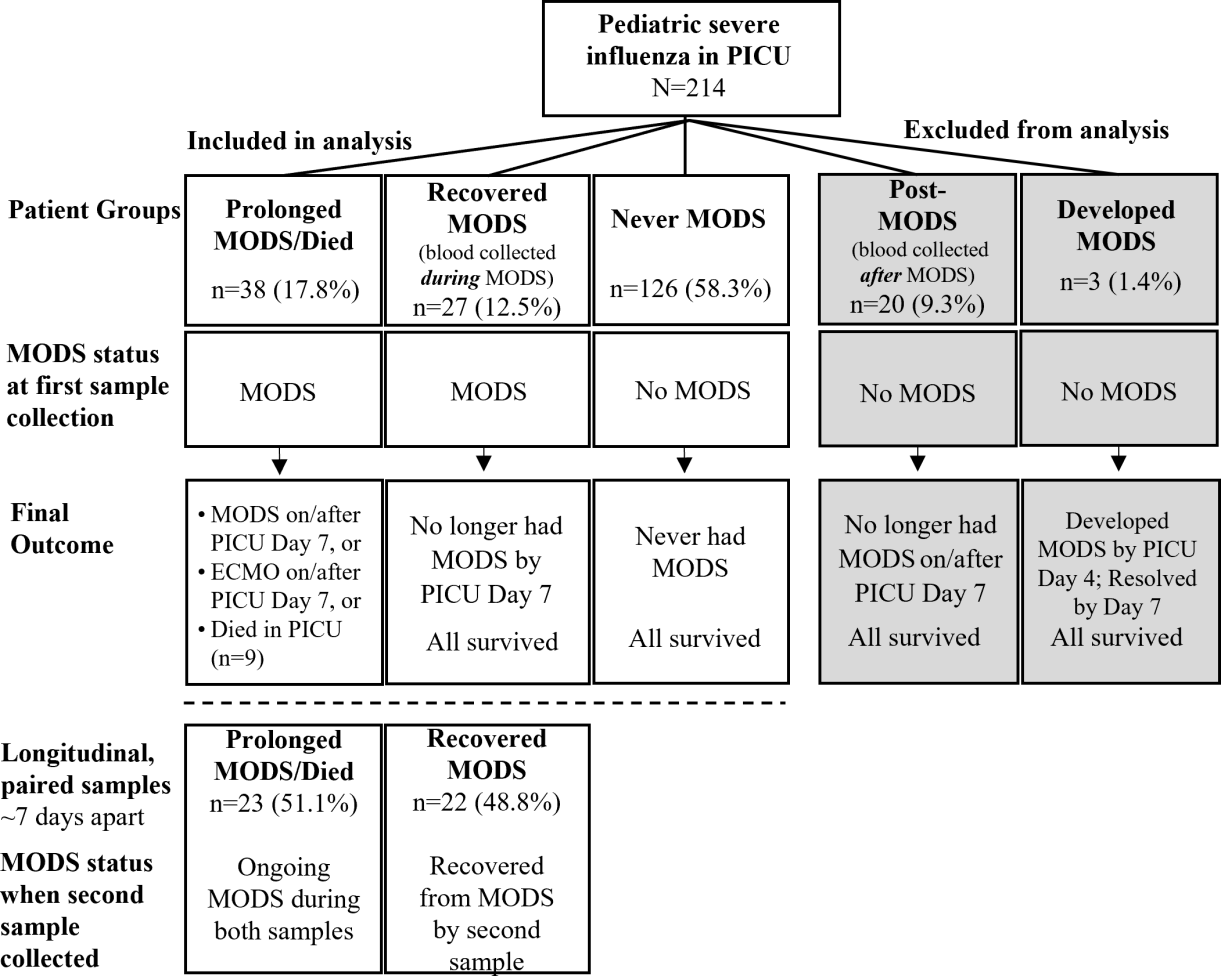
Children with influenza critical illness were stratified by their multiple organ dysfunction syndrome (MODS) status during blood collection and final outcome. Grey boxes indicate that patient groups were excluded from analysis. Longitudinal blood samples were collected from a subgroup of Prolonged and Recovered MODS patients in which a second blood sample was collected approximately 7 days after the first while still in hospital. PICU, Pediatric Intensive Care Unit. ECMO, Extracorporeal Membrane Support.

The Pediatric Sequential Organ Failure Assessment (pSOFA) score, is a validated measure of organ failure over time (maximum score of 24) that is positively correlated with mortality (11). It was used to determine each patient’s MODS status at the time of sample collection. MODS was defined as pSOFA ≥ 2 in at least two organ systems. See pSOFA Scoring in **Supplementary Material** for details. Each PICU patient was assigned to one of four MODS groups (**Figure 1**). “Prolonged MODS/Died**”** (subsequently referred to as “Prolonged MODS”) was defined as having MODS at the time of first blood collection plus one of the following: (a) MODS on/after PICU Day 7, and/or (b) ECMO on/after PICU Day 7, or (c) death while in hospital. Note only one death occurred on PICU Day 4; all others were after PICU Day 7. “Recovered MODS” was defined as having MODS at the time of first blood collection but survived with resolution of MODS by PICU Day 7. Patients who newly developed MODS within the first week after the first sample was collected were deemed as “Developed MODS”. Patients who never had MODS were assigned to the “Never MODS” control group. A subgroup of the Prolonged MODS and Recovered MODS patients had a second blood sample collected approximately seven days after their initial sample. Their MODS status was again determined on the day the samples were collected. **Figure 1** summarizes the cohort groups and definitions.

### Gene expression analyses

NanoString data were assessed for common quality control measures using the *NACHO* R package (12). Seven *a priori* defined housekeeping genes were assessed for frequency and degree of expression, and five of those were subsequently used to normalize the data with the geometric mean method *(B2M, DECR1, HPRT1, POLG, PPIB*). To identify potential genes that might distinguish the MODS patient groups, we utilized linear models as implemented by the *limma* package in R (13) and included log_10_ age and bacterial co-infections as covariates in each model. False Discovery Rate (FDR) was used to correct for multiple comparisons, with adjusted p-values <0.05 (or q values) considered significant. Paired longitudinal samples were analyzed using a linear model comparing Prolonged MODS vs Recovered MODS correcting for log_10_ age using FDR <0.05. The latter statistical approach was recently published (14) and is further outlined in the **Supplementary Material.**

## Results

We had high quality PAXgene RNA extracts from samples collected early in the PICU course on 191 pediatric patients (**Figure 1**). Of the final 191 patients included in the analysis, 38 had Prolonged MODS or died, 27 Recovered from MODS, 3 Developed MODS and 126 Never had MODS. Because only 3 patients Developed MODS, we excluded them from further analyses. Patients’ demographics and their clinical course are detailed in **Table 1** stratified by MODS category.

**Table 1:** Demographics and clinical course for severe pediatric influenza patient cohort.

Influenza cohort analyzed N=191	Prolonged MODS/Died	Recovered MODS	Never MODS	p-value*	p-value all 3 groups
n	38	27	126		
Age in years Median (IQR)	11.3 (6.2, 14.3)	6.9 (2.3, 9.8)	5.5 (1.7, 9.9)	0.01	<0.001
Sex, male	24 (63.2)	18 (66.7)	69 (54.8)	0.8	0.4
Hispanic	7 (18.4)	4 (14.8)	30 (23.8)	0.7	0.5
White or White-Hispanic	33 (86.8)	21 (77.8)	96 (76.2)	0.3	0.4
Black or African American	6 (15.8)	3 (11.1)	27 (21.4)	0.6	0.4
Mixed Ethnicity/Other/Unknown	3 (7.9)	4 (14.8)	10 (7.9)	0.4	0.6
Previously healthy	26 (68.4)	17 (63)	77 (61.1)	0.6	0.7
Influenza type					
A, H1	8 (21.1)	12 (44.4)	47 (37.3)	0.08	0.1
A, H3	9 (23.7)	4 (14.8)	35 (27.8)	0.6	0.4
B	16 (42.1)	8 (29.6)	18 (14.3)	0.4	<0.001
A+B	0	0	4 (3.2)	n/a	0.2
Not subtyped	5 (13.2)	3 (11.1)	22 (17.5)	1	0.6
Bacterial Co-Infection	26 (68.4)	8 (29.6)	28 (22.2)	0.002	<0.001
Viral Co-Infection	8 (21.1)	9 (33.3)	24 (19)	0.3	0.3
Hours from PICU admission to first sample Median, (IQR), Range	37.3 (23, 63.4) 8-201	25.6 (20, 48.1) 9-101	25.2 (16, 40) 2-110	0.2	0.02
pSOFA score at first collection Median (IQR)	9.5 (8, 11)	6 (5, 7.5)	0 (0, 2)	<0.001	<0.001
Steroids	27 (71.1)	18 (66.7)	76 (60.3)	0.7	0.5
Shock requiring vasopressors	23 (60.5)	16 (59.3)	11 (8.7)	0.9	<0.001
Mechanical Ventilation (invasive)	38 (100)	25 (92.6)	71 (56.3)	0.06	<0.001
ECMO	23 (60.5)	2 (7.4)	2 (1.6)	<0.001	<0.001
Hours in PICU Median (IQR)	481.5 (278, 920)	217 (162, 333.5)	86.5 (52, 155)	<0.001	<0.001
ALI/ARDS	35 (92.1)	21 (77.8)	19 (15.1)	0.1	<0.001
Died	9 (23.7)	0	0	0.02	<0.001
WBC† (10^3^/µL) Median (IQR)	3.3 (1.1, 8)	6.6 (3.3, 11.9)	10.1 (6.9, 14.1)	0.02	<0.001
ANC^‡^ (10^3^/µL) Median (IQR)	1.9 (0.4, 6.5)	4.9 (1.4, 10.3)	7.8 (4.3, 11.2)	0.03	<0.001
AMC^§^ (10^3^/µL) Median (IQR)	0.18 (0.07, 0.35)	0.41 (0.13, 0.76)	0.49 (0.23, 0.9)	0.06	<0.001
ALC^ll^ (10^3^/µL) Median (IQR)	0.56 (0.42, 0.92)	0.73 (0.48, 1.43)	0.98 (0.49, 1.9)	0.07	0.07

*p-value comparison between Prolonged and Recovered MODS. †Closest white blood cell (WBC) count to PAXgene blood collection; 166 out of 191 patients (86.9%) have WBC values; all missing WBC counts (n=25) are Never MODS. For WBC count timing, 84% were a median of 25.5 hrs (IQR 17.7, 46.3) prior to PAXgene draws (missing data for n=50). ^‡^ANC, Absolute neutrophil count missing for 2 Prolonged, 1 Recovered, 29 Never MODS. ^§^AMC, Absolute monocyte/macrophage count missing for 1 Prolonged, 2 Recovered, 29 Never MODS. ^ll^ALC, Absolute lymphocyte count missing for 1 Prolonged, 1 Recovered, 27 Never MODS. Non-adjusted p-values were determined using Kruskal-Wallis test for numerical data; Pearson Chi-Squared or Fisher’s Exact Test for categorical data. MODS, Multiple Organ Dysfunction Syndrome; IQR, Interquartile Range; pSOFA, Pediatric Sequential Organ Failure Assessment; ECMO, Extracorporeal Membrane Oxygenation; PICU, Pediatric Intensive Care Unit; ALI, Acute Lung Injury; ARDS, Acute Respiratory Distress Syndrome.

We compared the clinical characteristics and mRNA expression in blood collected during MODS from patients in the Prolonged MODS and Recovered MODS groups. They were also compared against Never MODS patients as a control group (**Table 1**). Patients with Prolonged MODS were older with a median age of 11.3 years (IQR 6.2, 14.3 years) and had a higher percent of bacterial co-infections (**Table S2**); therefore, we statistically adjusted analyses for these two variables. All three groups had a similar percentage of patients who were previously healthy, received steroids, and had other viruses co-detected in respiratory samples. Across cohorts, the first blood sample was obtained within a median of 27.1 hours of PICU admission (IQR 18.4, 44.8 hours). The time difference between collection of Prolonged MODS and Recovered MODS patients’ blood samples was not significantly different (p=0.2). For those who were not previously healthy, the most common pre-existing conditions were asthma (23% of n=191 cohort), neurological or neuromuscular developmental delay (5.8%), and seizure disorder not including simple febrile seizures (4.2%). Notably, 8 of 9 Prolonged MODS patients who died had no underlying conditions, and one had mild asthma. The Prolonged MODS group had a longer duration of PICU stay, higher incidence of invasive mechanical ventilation and shock requiring vasopressors, and increased frequency of ARDS diagnosis and/or ECMO support. Influenza B was also more common in this group. White blood cells and neutrophil counts were significantly lower in Prolonged MODS patients compared to Recovered MODS patients. The numbers of monocyte and lymphocytes were not significantly different.

The six organ systems identified as dysfunctional in patients with either Prolonged MODS or Recovered MODS are listed in **Table 2**. These patients had very similar percentages of their respiratory, cardiovascular, and neurologic systems affected at the time of the first blood collection. Prolonged MODS patients sustained a higher percent of cases with coagulation, hepatic, and renal dysfunction. The majority of the Recovered MODS patients (85.2%) only had two of the six organ systems affected while 68.4% of Prolonged MODS patients had three or more affected.

**Table 2:** Number of dysfunctional organ systems in patients with Prolonged MODS/Died or Recovered MODS at the time of the first blood collection.

Organ Systems Dysfunction* n (%)	Prolonged MODS/Died	Recovered MODS
N	38	27
Respiratory	35 (92.1)	26 (96.3)
Coagulation	25 (65.8)	7 (25.9)
Hepatic	10 (26.3)	1 (3.7)
Cardiovascular	32 (84.2)	23 (85.2)
Renal	11 (28.9)	2 (7.4)
Neurologic	3 (7.9)	2 (7.4)
2 systems	12 (31.6)	23 (85.2)
3 systems	17 (44.7)	2 (7.4)
≥4 systems	9 (23.7)	2 (7.4)

*Evaluation of organ dysfunction was based on the pediatric Sequential Organ Failure Assessment (pSOFA) (11).

### Neutrophil degranulation is significantly associated with Prolonged MODS and/or death in pediatric severe influenza LRTI

**Figure 2:**
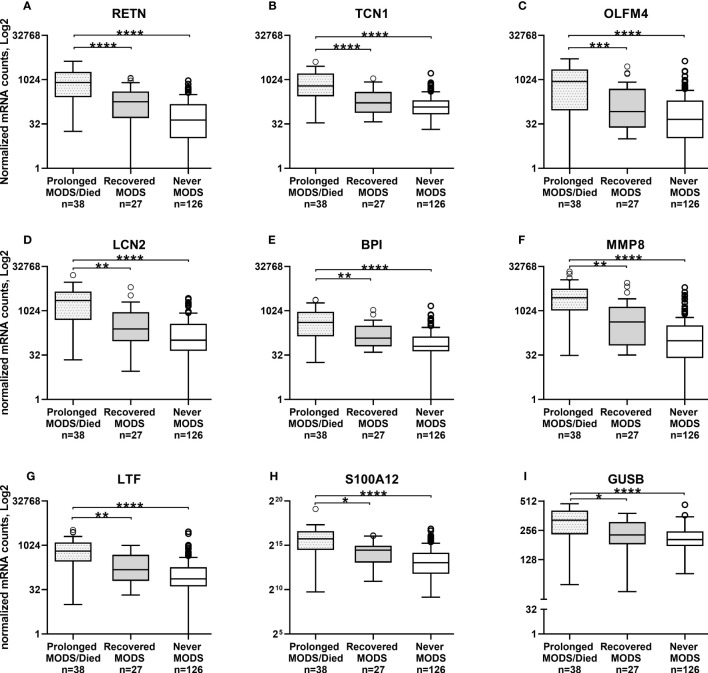
Whole blood collected during MODS revealed significantly increased mRNA expression of nine genes associated with neutrophil degranulation in pediatric patients who had Prolonged MODS or Died (n=38) compared to patients that Recovered from MODS (n=27). Never MODS was included as an influenza positive PICU control (n=126). After adjustment for multiple comparisons, transcription levels between Recovered vs Never MODS did not have any statistically differences. Note the different y-axis for H and I. ****q<0.0001, ***q ≤ 0.001, **q ≤ 0.01, *q<0.05. All Tukey box plots represent the median (center line of the box), 25% IQR (Q1, bottom line of the box), 75% IQR (Q3, top line of the box), error bars are the minimum and maximum values within 1.5x the Q1 and Q3 quadrants, outliers indicated by circles. MODS, Multiple Organ Dysfunction Syndrome. **(A)** RETN, Resistin. **(B)** TCN1, Haptocorin. **(C)** OLFM4, Olfactomedin 4. **(D)** LCN2, Lipocalin 2. **(E)** BPI, Bactericidal Permeability Increasing Protein. **(F)** MMP8, Matrix Metallopeptidase 8. **(G)** LTF, Lactotransferrin. **(H)** S100A12, S100 Calcium Binding Protein A12. **(I)** GUSB, Glucuronidase Beta.

**Figure 3:**
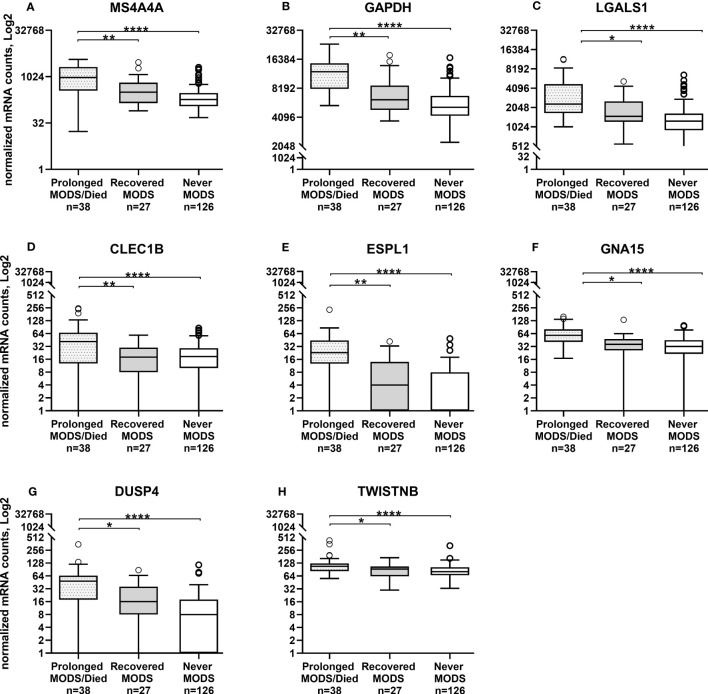
Whole blood collected during MODS showed significantly increased mRNA expression of eight genes not directly associated with neutrophil degranulation in patients that had Prolonged MODS or Died (n=38) compared to those that who recovered from MODS (n=27). Never MODS was included as an influenza positive PICU control (n=126). After adjustment for multiple comparisons, transcription levels between Recovered vs Never MODS did not have any statistically differences. ****q<0.0001, **q ≤ 0.01, *q<0.05. MODS, Multiple Organ Dysfunction Syndrome. **(A)** MS4A4A, Membrane Spanning 4-Domains A4A. **(B)** GAPDH, Glyceraldehyde-3-Phosphate Dehydrogenase. **(C)** LGALS1, Galectin 1. **(D)** CLEC1B, C-Type Lectin Domain Family 1 Member B. **(E)** ESPL1, Separin. **(F)** GNA15, G protein subunit alpha 15. **(G)** DUSP4, Dual specificity phosphatase 4. **(H)** TWISTNB, RNA polymerase I subunit F.

Comparison of the quantity of mRNA transcripts (interpreted as mRNA expression levels) between patients with Prolonged MODS (n=38) and Recovered MODS (n=27) identified 17 genes with significantly increased expression in those with Prolonged MODS patients. Nine of these genes are involved specifically with neutrophil degranulation (*RETN, TCN1, OLFM4, LCN2, BPI, MMP8, LTF, S100A12, GUSB*) (**Figure 2** and **Table 3**), whereas the other eight genes (*MS4A4A CLEC1B, GAPDH*, *LGALS1, ESPL1, GNA15, DUSP4*, and *TWISTNB)* are involved in various cellular functions: metabolism, cell proliferation and differentiation, blood coagulation, and/or cell cycling (**Figure 3** and **Table 4**). Three of the genes involved in neutrophil degranulation, *RETN, TCN1*, and *OLFM4*, had the largest differences among all 17 genes (q ≤ 0.001). Absolute neutrophil counts from Prolonged MODS and Recovered MODS patients showed a significant inverse relationship with all neutrophil degranulation mRNA transcript levels except for *OLFM4* and *BPI*, respectively (**Figure S1**). There was no correlation between neutrophil counts and Never MODS patients’ mRNA levels.

**Table 3:** Nine genes associated with neutrophil degranulation that had significantly increased mRNA levels in patients with Prolonged MODS/Died compared to Recovered MODS.

Gene	Gene Full Name/Alias	Log2 fold change	P value	FDR adjusted P value*	References
**RETN**	Resistin, Cysteine-Rich Protein Precursor 1	777.914	9.51E-08	0.00005	(8, 15, 16)
**TCN1**	Haptocorin, Transcobalamin 1, Vitamin B12 Binding Protein, R Binder Family	613.987	4.48E-07	0.0001	(8, 15)
**OLFM4**	Olfactomedin 4, 2’-5’-Oligoadenylate Synthetase 2, G-CSF-Stimulated Clone 1 Protein, GC1	923.158	5.36E-06	0.0008	
**LCN2**	Lipocalin 2, Neutrophil Gelatinase-Associated Lipocalin, 25 KDa Alpha-2-Microglobulin-Related Subunit Of MMP-9	2048.845	6.29E-06	0.0008	
**BPI**	Bactericidal Permeability Increasing Protein, B Cell CLL/Lymphoma 3	377.357	1.41E-05	0.001	
**MMP8**	Matrix Metallopeptidase 8, Neutrophil Collagenase	2621.675	3.13E-05	0.002	
**LTF**	Lactotransferrin, Neutrophil Lactoferrin	569.083	0.000111134	0.005	(15, 17)
**S100A12**	S100 Calcium Binding Protein A12, Calgranulin-C, ENRAGE	38819.741	0.000426456	0.02	(18)
**GUSB**	Glucuronidase Beta	66.579	0.000427102	0.02	(15, 19)

*q value.

**Table 4:** Eight genes not associated with neutrophil degranulation that had significantly increased mRNA levels in patients with Prolonged MODS/Died compared to Recovered MODS.

Gene	Gene Full Name/Alias	Log2 fold change	P value	FDR adjusted P value*	References
**MS4A4A**	Membrane Spanning 4-Domains A4A, CD20L1	599.520	3.57E-05	0.002	(20, 21)
**GAPDH**	Glyceraldehyde-3-Phosphate Dehydrogenase	3424.388	4.39E-05	0.002	(22)
**CLEC1B**	C-Type Lectin Domain Family 1 Member B, CLEC-2	34.186	1.84E-05	0.002	(23, 24)
**ESPL1**	Extra Spindle Pole Bodies Like 1, Separin, Cysteine Protease	18.204	0.000118	0.005	(25, 26)
**GNA15**	G protein subunit alpha 15	21.078	0.000468	0.02	(9, 27)
**LGALS1**	Galectin 1, Lactose-Binding Lectin 1	1180.432	0.000979	0.03	(28, 29)
**DUSP4**	Dual specificity phosphatase 4	24.236	0.001446	0.05	(30, 31)
**TWISTNB**	Twist Neighbor, POLR1F, RNA polymerase I subunit F, DNA-directed RNA polymerase I subunit RPA43	35.016	0.001688	0.05	(32)

*q value.

In children who never had MODS, all were admitted to the PICU due to influenza-related respiratory system involvement. Differentially expressed genes between the Recovered MODS and Never MODS patients were not significant after adjustment for multiple comparisons. Never MODS patients showed significant gene expression differences compared with Prolonged MODS in 94 genes (**Table S3A**); these included the 17 genes in **Figures 1**, **2** differentially expressed between the Prolonged and Recovered MODS groups. Nine genes are listed in **Table S3B** that were most similar between the Prolonged and Never MODS groups.

### Although neutrophil degranulation transcripts were associated with a Prolonged MODS outcome, differential expression over time of multilineage immune cell activation-related genes were associated with clinical recovery

**Figure 4:**
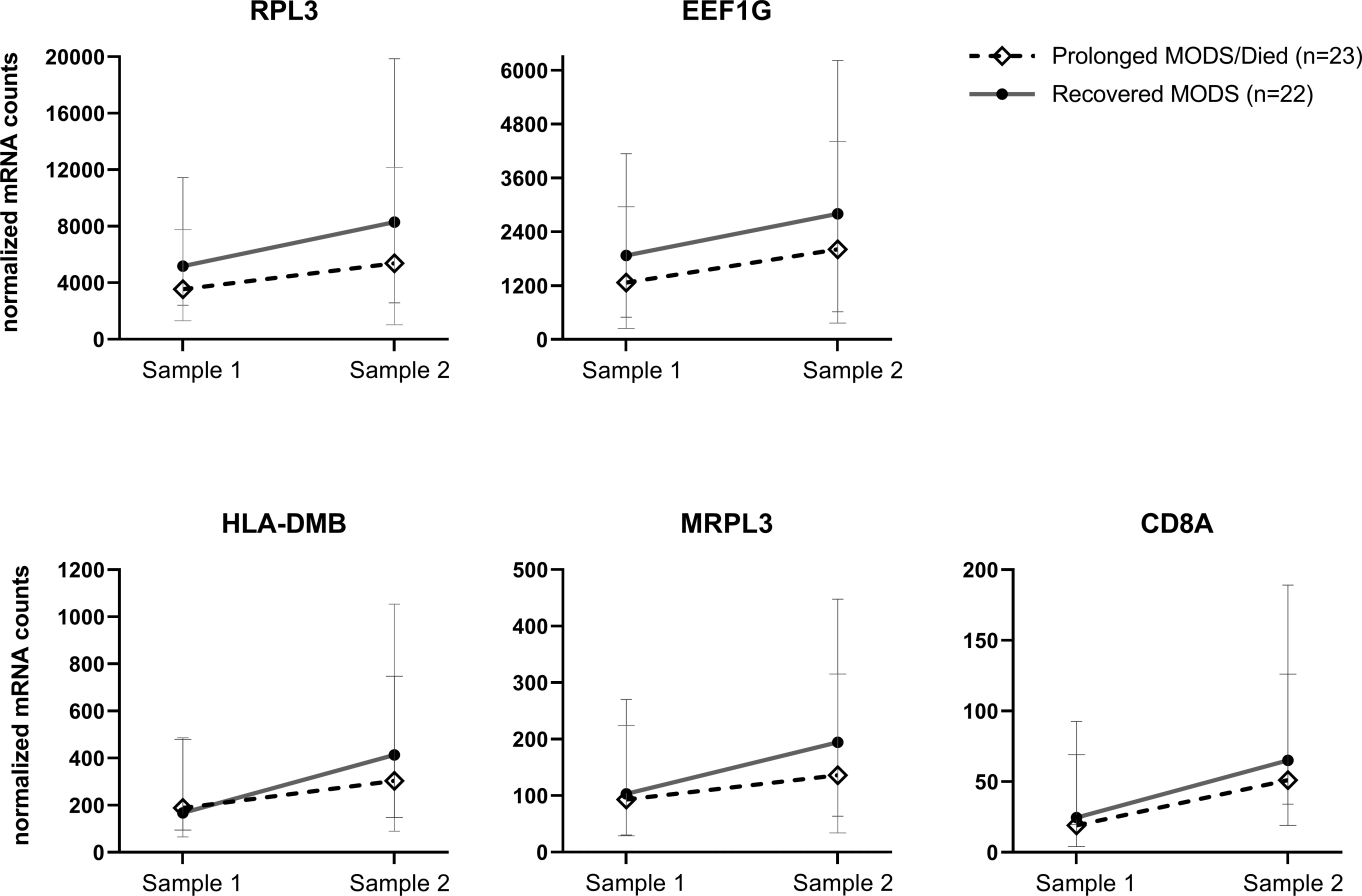
Longitudinal, paired blood samples from Prolonged MODS/Died versus Recovered MODS patients: Five genes whose mRNA expression significantly differed between their first and second samples obtained approximately 7 days apart. Median normalized mRNA levels are plotted for each subgroup with 95% confidence intervals of the median. These genes showed a differential change over time between the first, early blood collection, Sample 1, and Sample 2 collected approximately 7 days later from Prolonged MODS/Died patients (n=23, staggered line, ◊ median expression) and Recovered MODS patients (n=22, solid line, ● median expression), FDR q<0.05. Note: for each graph, y-axis linear scales vary. RPL3, Ribosomal protein L3. EEF1G, Eukaryotic Translation Elongation Factor 1 Gamma protein. HLA-DMB, Major histocompatibility complex, class II, DM beta. MRPL3, Mitochondrial Ribosomal Protein L3. CD8A, T-cell surface glycoprotein CD8 alpha chain.

A second PAXgene sample was collected from 81 patients a median of 6.9 days (IQR 6.8, 7.1) after the first sample collection. The Never MODS subgroup was excluded as the focus was to compare biological mRNA levels in those that had MODS at one time. We were able to collect a second sample from 23 patients who still had Prolonged MODS and compare them to 22 patients who had Recovered from MODS (which included seven previous Prolonged MODS patients who had recovered by their second sample). The mRNA expression in early versus later samples was analyzed (**Table 5**). Five genes (*RPL3, EEF1G, HLA-DMB, MRPL3* and *CD8A*) exhibited significant differences in gene expression when comparing paired longitudinal samples collected approximately one week apart from Recovered MODS patients and those with Prolonged MODS. To visualize the change of expression in each of these genes in the Prolonged MODS and Recovery groups, **Figure 4** shows the trajectory of each of these gene products between sample 1 (the first, earliest PAXgene blood RNA sample) and sample 2 (PAXgene blood RNA collection obtained approximately seven days after the first) (q<0.05). The proportion of patients in each MODS phenotype with increased or decreased mRNA expression is shown in **Figure S2**. *RPL3, MRPL3*, and *HLA-DMB* expression increased in 78.3% of Prolonged MODS patients, while *EEF1G* and *CD8A* decreased in nearly a third of the group. *HLA-DMB* and *CD8A* increased in nearly all (95.4%) Recovered MODS patients. *RPL3, MRPL3*, and *EEF1G* also increased in the majority (90.9%) of the Recovered MODS group.

**Table 5:** Five genes showed significantly different mRNA expression in longitudinal paired samples when comparing Prolonged MODS and Recovered MODS over time.

Gene	Gene Full Name/Alias	Prolonged MODS mean contrasts	Prolonged MODS SD contrast	Recovered MODS mean contrasts	Recovered MODS SD contrast	P value null	P value alt	Ref
RPL3	Ribosomal protein L3	-2754.08	4599.48	-3776.05	3478.23	9.51E-05	8.77E-73	(33, 34)
EEF1G	Eukaryotic Translation Elongation Factor 1 Gamma protein; EF1G; GIG35	-702.35	1376.84	-1034.14	856.87	4.14E-05	4.87E-62	(33, 34)
HLA-DMB	Major histocompatibility complex, class II, DM beta	-144.83	239.94	-269.27	183.75	1.31E-04	8.71E-45	(32)
MRPL3	Mitochondrial Ribosomal Protein L3	-43.74	93.65	-73.41	63.82	1.08E-04	6.02E-35	(35–38)
CD8A	CD8a Molecule, CD8 antigen, alpha polypeptide (p32), T-cell surface glycoprotein CD8 alpha chain	-31.57	60.8	-48.45	47.67	3.52E-06	1.15E-25	(39)

Contrasts, the difference in measurements between two timepoints; SD, standard deviation; Alt, alternative; Ref, References.

## Discussion

In this national cohort of over 200 children with severe influenza LRTI admitted to the PICU, development of prolonged MODS was a life-threatening and sometimes fatal complication associated with high morbidity (4). Using a targeted gene panel of 469 mRNA transcripts on whole blood samples collected from these severely ill patients early in their PICU course, we identified increased expression of multiple genes in the neutrophil degranulation pathway in children that went on to have poor clinical outcomes. These transcripts (including *RETN, TCN1, OLFM4, LCN2, BPI, MMP8, LTF, S100A12*, and *GUSB*) distinguished children with Prolonged MODS (and those who later died) from those who had MODS at the time of sample collection but recovered within 7 days and from those who never developed MODS. Serial samples collected approximately a week later did not show major differential changes over time in these same genes between the Prolonged MODS and Recovered MODS groups. Instead, results showed increased longitudinal differential expression levels in the Recovered MODS subgroup of gene transcripts known to be involved in adaptive immunity, the innate-adaptive immune interface, or ribosome activation.

Despite significant differences in the immunological development and correlates of risk and protection between children and adults, our neutrophil degranulation signature is consistent with the findings of prior studies in adult patients with severe LRTI or sepsis with ARDS. Some of the genes from these publications were incorporated into our mRNA panel and revealed a similar upregulation in our Prolonged MODS group. In 2018, Dunning and colleagues (15) using a custom microarray on samples collected within approximately four days of symptom onset in 31 mechanically ventilated UK adults hospitalized for severe influenza with and without bacterial coinfection, reported increased expression of seven of these same neutrophil degranulation genes (*RETN, TCN1, OLFM4, LCN2, BPI, MMP8*, and *LTF*). In our cohort, we statistically adjusted for the presence of confirmed bacterial coinfections and did not report them separately. Kangelaris and colleagues (8) previously reported that *RETN, TCN1, OLFM4*, *LCN2, BPI*, and *MMP8* neutrophil degranulation genes were upregulated in the peripheral blood of 28 older adults during sepsis-related ARDS (72% due to pneumonia/lung aspiration) compared to those with sepsis without lung involvement. Similarly, over 92% of our Prolonged MODS cohort also suffered with acute lung injury (ALI) or ARDS. Liu and colleagues also identified these same six genes in a Chinese cohort as potential biomarkers for severe influenza LRTI in adults (40). Another study reported that the upregulation of neutrophil mRNA transcripts were strongly associated with adverse outcomes from severe influenza virus LRTI in critically ill adults admitted to the ICU of which 32% had MODS, 16% had a bacterial co-infection, and 20% died (41). The same adult cohort was further validated by Zerbib, et al, who found that severe influenza was associated with various neutrophil pathways, including degranulation (42).

Additional publications describe some of the same genes, albeit individually, as being associated with illness severity. In a recent report, adults hospitalized with coronavirus disease 2019 (COVID-19), had increased serum resistin protein levels (derived from *RETN*) associated with invasive ventilation and poor prognosis (16). A different study also confirmed increased *GUSB* in adult ICU sepsis patients (19). Of note, although *GUSB* has been used as a housekeeping gene in expression studies (43), we and others show that it can function as a measure of disease severity for influenza and sepsis. In our study, *GUSB* ultimately showed significantly different expression levels in the Prolonged MODS compared to Recovered MODS groups and was therefore excluded as a housekeeping gene.

The Prolonged MODS cohort overall had lower neutrophil counts, therefore the increase of neutrophil degranulation mRNA transcripts in this cohort is not explained by increased neutrophil counts. This inverse relationship between higher expression of neutrophil degranulation genes with lower neutrophil counts in more severely ill patients was also reported in severe sepsis/ARDS patients (8). Neutropenia in a critically ill patient could be explained by lack of neutrophil production, sequestration, or activation induced cell death (44–46). Our finding that 8 of 9 neutrophil degranulation genes were negatively correlated with neutrophil count identifies a potential gene signature that may be useful for characterizing the latter cause of neutropenia in infected patients with Prolonged MODS.

Although these nine neutrophil degranulation mRNA transcripts have various end-point functions, collectively, they support a strong association of active neutrophil degranulation pathways with prolonged disease severity evidenced by multiorgan dysfunction. In our study, over 92% of patients with MODS had respiratory dysfunction. Other affected organs were highly variable per patient making difficult to speculate the involvement of each. The consistency of this neutrophil signature in adult influenza cohorts and our own data suggests it may represent an initial key pathway for severe outcomes particularly in respiratory disease.

Previous findings suggest that obtaining specimens within 24 hours of admission is necessary to predict outcomes based on neutrophil-mediated expression (8); however, the general timeframe between illness onset and PICU admission is largely variable. Our results of influenza-infected children with Prolonged MODS revealed that neutrophil-related mRNA transcription remained upregulated for a median of 37.3 hours (IQR 23, 63.4 hours), similar to what Dunning, et al, and Tang, et al, discovered in adults (15, 41). However, there was no significant difference in the change in expression of the neutrophil gene signature over time (i.e. a week later).

To further differentiate gene expression in blood collected during MODS, there were eight additional genes not directly related to neutrophils that were upregulated early on in the Prolonged MODS versus Recovered MODS groups. The first, *MS4A4A*, codes for a novel cell surface marker for alternatively activated macrophages and plasma cells (20) and was previously reported as being significantly increased in pediatric sepsis (21). *ESPL1*, encodes a key protease needed to initiate anaphase, and has not been previously reported as being elevated in influenza or MODS; however, there are studies suggesting a role in cell cycle dysregulation during influenza infections (25). In blood immune cells from healthy adults, *ESPL1* mRNA is transcribed in low quantities in T- and B-cells but largely stems from short-lived plasma cell precursors (26). Its increase in the Prolonged MODS group could indicate an attempt at proliferation by adaptive immune cells to help fight infection. Similarly, *CLEC1B* encodes for a transmembrane protein on platelets, granulocytes, and myeloid cells and has an anti-inflammatory role during bacterial-induced ARDS in mice (23). *CLEC1B* was also shown to be increased in asymptomatic SARS-CoV-2 adults approximately two weeks after their exposure, suggestive of a role in viral infection recovery (24). Although *LGALS1*, *GNA15*, *DUSP4*, and *TWISTNB* were also upregulated in the Prolonged MODS versus Recovered MODS groups, their differentiation was less pronounced. However, they may have prognostic value deserving of further study. They are explored further in the **Supplementary Material**.

In the subset of children with MODS with longitudinal samples, five mRNA transcripts *RPL3, EEF1G, HLA-DMB, MRPL3*, and *CD8A*, differed in expression between those with Prolonged MODS and Recovered MODS. *RPL3* and *EEF1G*, which enable different ribosomal-related enzyme functions, are commonly downregulated during *initial* acute influenza infection (33). Likewise, we also found that they were similarly downregulated in early samples compared to later ones. In a separate pediatric study, *EEF1G* mRNA expression and specifically *RPL31*, were reported to be decreased in PBMCs from influenza-infected children following hospitalization and could be used to distinguish between influenza and bacterial infections (with and without lung involvement) (34). In our longitudinal cohort, >90% of the Recovered MODS subgroup showed increased *RPL3* and *EEF1G* expression at their second time point suggesting a return to normal cellular function since these genes are vital transcriptional components of ribosome function and protein synthesis. Another ribosome associated gene, *MRPL3*, which encodes a mitochondrial ribosomal protein, facilitates the synthesis of mitochondrial proteins needed for oxidative phosphorylation (35), an essential metabolic process for the development and function of effector CD8^+^ T cells (36, 37). Interestingly, it has also been associated with high altitude hypoxia (38) suggesting its upregulation in the Recovered MODS group may be helping increase oxygenation utilization. Finally, two genes important in either directly or indirectly activating the adaptive immune response, *CD8A* and *HLA-DMB*, showed increased expression in 95% of the Recovered MODS subgroup at their second time point, suggesting the importance of increased recovery of the function of the innate-adaptive immune interface may be key for recovery of organ function. It could also represent an expansion of CD8+ T cells in the blood, indicating a rebound from the lymphopenia that characterizes many severe viral and bacterial infections. In a separate study, a 14-dataset analysis of adult and pediatric sepsis spanning 8 different countries, the decrease of *HLA-DMB* expression in whole blood was observed soon after a sepsis diagnosis in the most severe cases (32). This was also true in our study in the first blood sample from children with Prolonged MODS and Recovered MODS. However, at their second timepoint, those who recovered from MODS demonstrated significantly increased *HLA-DMB*, potentially signifying an enhanced ability of those subjects’ innate immune cells to perform MHC class II-mediated antigen presentation.

We have previously shown that functional impairment of circulating leukocytes, as measured by reduced ability to produce pro-inflammatory cytokines when whole blood is stimulated *ex vivo* using lipopolysaccharide or poly (I:C), was associated with increased risks for mortality, prolonged organ dysfunction, and prolonged hypoxemic respiratory failure in critically ill children with influenza (6). Of note, in this study, all influenza-positive children admitted to the PICU, regardless of MODS status, had similar, abundant interferon receptor expression levels (*IFNαR1*, *IFNγR1*) at their first time point (data not shown). Many of the other interferon transcripts, such as *IFNα2, IFN1/13*, and *IFNγ*, had very low mRNA counts suggesting these particular influenza viral responses had likely diminished but not before they induced a high level of *IFITM1* (47) which was equally maintained in the Prolonged MODS and Never MODS groups despite the variable complications. The results of the current study suggest that phenomena such as neutrophil degranulation may occur concurrently with down-regulation of innate immune responsiveness.

## Study strengths and limitations

The strengths of this study included multicenter enrollment across the U.S. and extensive phenotyping of patients over eight influenza seasons. The study’s longevity (and therefore familiarity at PICU sites) has enabled the cohesive capture of numerous MODS cases at early time points. Also, capturing two sample time points seven days apart provided an insight to the transcriptional activity trend over time and differentiated patients that recovered faster. Our study also has some limitations. First, this study is prognostic and only establishes associations and not causality. Second, the protein level produced and/or released into the extracellular environment was not evaluated in this study, as our evaluation of samples was limited to mRNA expression based on *a priori* selection of genes. As is the case with any gene expression study of whole blood, highly expressed target genes, such as S100A12, may obscure quantification. Third, although WBC counts were determined for most of the cohort, the information was reliant on hospital site clinical sample lab results, which were obtained at variable times – many were one day prior to the research sample being obtained. We were unable to measure WBC on the research samples due to limitations on how much blood could be obtained from children and appropriate instrumentation/staff for conducting differential counts at multiple locations. Therefore, neither WBC or neutrophil counts were statistically controlled for as confounders as not all data points were available. However, for all samples, RNA content from whole blood is derived almost entirely from white blood cells. Thus, gene expression was normalized by (1): standardizing the amount of RNA tested and (2) normalizing all expression data to the same housekeeping genes. Fourth, vaccination status was self-reported in this cohort and therefore not verified. However, an average of 36% of the parents interviewed responded that their child had received the seasonal influenza vaccine prior to hospital admission, which was similar for all MODS groups. Fifth, our multicenter cohort included only three children who developed MODS after blood was collected. To be able to analyze this patient group, it would help to broaden the influenza cohort by preemptively collecting blood samples while in the emergency room or ward.

## Conclusion

In conclusion, children with influenza critical illness who endured prolonged MODS or died had multiple blood mRNA transcripts associated with neutrophil degranulation shortly after PICU admission compared to those that recovered from MODS. We show similar findings that were reported in prior transcriptional studies on sepsis and/or influenza-infected hospitalized adults. Although prognostic, neutrophil pathways did not change differentially across the Prolonged versus Recovered MODS groups in the subset of children whose samples were obtained a week later. Instead, genes involved with adaptive immunity and ribosomal protein synthesis were higher in those who recovered from MODS. Cumulatively, these data aid in understanding the pathophysiology of children with concurrent severe influenza LRTIs and MODS. Importantly, it provides gene expression data that may help to develop prognostic approaches to improve monitoring and prompt intervention for these complex cases and, based on prior studies, may be applicable across multiple age cohorts. These findings require validation in other cohorts of critically ill children.

## Data availability statement

The original contributions presented in the study are publicly available. This data can be found here: https://www.ncbi.nlm.nih.gov/geo/query/acc.cgi?acc=GSE236877.

## Ethics statement

The studies involving human participants were reviewed and approved by Institutional Review Boards at each hospital site. Written informed consent to participate in this study was provided by the participants’ legal guardian/next of kin.

## Author contributions

TN has first authorship. AGR and PGT devised the study and as authors contributed equally to this work and share senior authorship. AGR was the principal investigator. MWH, PMM, KMT, BM, LLL, SLW, RH, AJS, NPP, NJT, RGG, RCS, SM, BMC, NZC, KGA, DWT, PM, SCK, SLS, and AGR contributed to patient enrollment, sample collection, and clinical data acquisition. MMN collated and interpreted clinical data. ST assisted in gene target selection. TN conducted the experiments. JCC, GH, and CL conducted statistical analysis. TN and AGR interpreted the data and wrote the first draft of the manuscript. All authors contributed to the article and approved the submitted version.

## Group member of PALISI PICFLU study investigators

Children’s of Alabama, Birmingham (Michele Kong, MD, Kate Sewell, RN, BSN, Mary Jane O’Dell); Phoenix Children’s Hospital, Phoenix, Arizona (David Tellez, MD, Courtney Bliss, MS, Aimee Labell, MS, RN, Danielle Liss, BA, Ashely L. Ortiz, BA); Banner Children’s/Diamond Children’s Medical Center, Tucson, Arizona (Katri Typpo, MD, Jen Deschenes, MPH, Connor P. Kelley, MPH); Arkansas Children’s Hospital, Little Rock (Ronald C. Sanders, Jr, MD, MS, Glenda Hefley, RN, MNsc); Children’s Hospital of Los Angeles, California (Barry Markovitz, MD, Jeff Terry, MBA, Rica Sharon P. Morzov, RN, BSN, CPN); Children’s Hospital of Orange County, Orange, California (Adam Schwarz, MD, Nick Anas, MD, Chisom Onwunyi, RN, BSN, MPH, CCRP, Cathy Flores, RN, Stephanie Osborne, RN, CCRC, Tiffany Patterson, BS, CRC, Ofelia Vargas-Shiraishi, BS, CCRC); UCSF Benioff Children’s Hospital Oakland, California (Heidi Flori, MD, Natalie Cvijanovich, MD, Becky Brumfield, RCP, Julie Simon, RN); UCSF Benioff Children’s Hospital San Francisco, California (Patrick McQuillen, MD, Anil Sapru, MD, Anne Mckenzie, RN, Maureen Convery, BS, Victoria Lo, BA); Children’s Hospital Colorado, Aurora (Peter M. Mourani, MD, now at Arkansas Children’s Research Institute; Aline B. Maddux, MD, MSCS, Angela Czaja, MD, Valeri Batara Aymami, RN, MSNCNS, Susanna Burr, CRC, Megan Brocato, CCRC, Stephanie Huston, BS, RPSGT, CRC, Emily Jewett, Sandra B. Lindahl, RN, Danielle Loyola, RN, BSN, MBA, Yamila Sierra, MPH); Yale-New Haven Children’s Hospital, New Haven, Connecticut (John S. Giuliano Jr., MD, Joana Tala, MD, Lisa Palmieri); Holtz Children’s Hospital, Miami, Florida (Gwenn McLaughlin, MD); Children’s Healthcare of Atlanta at Egleston, Atlanta, Georgia (Keiko M. Tarquinio, MD, Matthew Paden, MD, Chee-Chee Manghram, Stephanie Meisner, RN, BSN, CCRP, Cheryl L. Stone, RN, Rich Toney, RN, Kaitlin ChanceJones, RN); The University of Chicago Medicine Comer Children’s Hospital, Chicago, Illinois (Neethi P. Pinto, MD, MS, Andrea DeDent, PhD, Chiara Rodgers MPH, CCRC); Ann and Robert H. Lurie Children’s Hospital of Chicago, Illinois (Bria M. Coates, MD, Avani Shukla, MS, MA, CCRP, Laura Campbell, BS); Norton Children’s Hospital, Louisville, Kentucky (Janice Sullivan, MD, Vicki Montgomery, MD, FCCM, Tracy Evans, RN, Kara Richardson, RN, Jennifer Nason, Kristen Lee); Boston Children’s Hospital, Boston, Massachusetts (Adrienne G. Randolph, MD, MS, Margaret M. Newhams, MPH, Anna A. Agan, MPH, Tanya Novak, PhD, Suden Kucukak, MD, Takuma Kobayashi, BS); Johns Hopkins Children’s Center, Baltimore, Maryland (Melania Bembea, MD, MPH, Sapna Kudchadkar, MD, Elizabeth D. White, RN, CCRP); Children’s Hospital and Clinics of Minnesota, Minneapolis (Stephen C. Kurachek, MD, Angela A. Doucette, CCRP, Erin Zielinski, MS, Glennie Mihalovic); St Louis Children’s Hospital, St Louis, Missouri (Mary E. Hartman, MD, MPH, Julie Bubeck Wardenburg, MD, Allan Doctor, MD, Rachel Jacobs, BA, Shivan Shetty, MPH, Stephanie Schafer); Children’s Hospital and Medical Center, Omaha; University of Nebraska Medical Center, Omaha, Nebraska (Sidharth Mahapatra, MD, PhD, Edward Truemper, MD, Machelle Dawson, RN, BSN, MEd, CCRC, Lucinda R. Kustka, RN, BSN, Kristin Malone, Melanie Schrack); Golisano Children’s Hospital, Rochester, New York (Kate G. Ackerman, MD, MBA, Louis Eugene Daugherty, MD, Laurel Baglia, PhD); Nationwide Children’s Hospital, Columbus, Ohio (Mark W. Hall, MD, Kristin Greathouse, BSN, MS, Lisa Steele, RN, BSN, CCRN, Josey Hensley, RN, BSN); University Hospitals Rainbow Babies and Children’s Hospital, Cleveland, Ohio (Steven L. Shein, MD, Ramon Adams, BA, Susan Bergant, Eloise Lemon, MSN, MHA, RN, CCRC, Lisa Petersen, RN, BSN, Kathryn DiFrancesco, BA, CCRP); Akron Children’s Hospital, Akron, Ohio (Ryan Nofziger, MD, FAAP, Heather Anthony, RN); Children’s Hospital of Philadelphia, Pennsylvania (Scott L. Weiss, MD, now currently at Nemours Children’s Hospital Delaware; Julie C. Fitzgerald, MD, PhD, Mark Helfaer, MD, Jenny L. Bush, RNC, BSN, Mary Ann Diliberto, RN, Jillian Egan, RN, Brooke B. Park, RN, BSN, Martha Sisko, RN, BSN, CCRC); Penn State Children’s Hospital, Hershey, Pennsylvania (Neal J. Thomas, MD, Debra Spear, RN); Texas Children’s Hospital, Houston (Laura L. Loftis, MD, Nancy Jaimon, RN, MSN-Ed, Ursula Kyle, MS, Daphne Peoples, Amica Onyemeh-Sea); Dell Children’s Medical Center of Central Texas, Austin, Texas (Renee Higgerson, MD, LeeAnn Christie, RN, PhD); University of Texas Southwestern Medical Center at Dallas (Cindy Darnell Bowens, MD) Children’s Hospital of Wisconsin, Milwaukee, (Rainer G. Gedeit, MD, Briana E. Horn, Kate Luther, MPH, Katherine Siegel, Kathy Murkowski, RRT, CCRC).

## References

[1] Ruf BR Knuf M. The burden of seasonal and pandemic influenza in infants and children. Eur J Pediatr (2014) 173(3):265–76. doi: 10.1007/s00431-013-2023-6 PMC3930829 23661234

[2] FluSurv-NET influenza hospitalization surveillance network. Laboratory-Confirmed Influenza Hospitalizations: Centers for Disease Control and Prevention (2022) Atlanta, GA, United States: FluSurv-NET: Influenza Hospitalization Surveillance Network. Available at: https://gis.cdc.gov/grasp/fluview/fluhosprates.html.

[3] FluSurv-NET: influenza-associated pediatric mortality surveillance network. Influenza Associated Pediatric Deaths: Centers for Disease Control and Prevention (2022). Atlanta, GA, United States: Available at: https://gis.cdc.gov/GRASP/Fluview/PedFluDeath.html.

[4] Watson RS Crow SS Hartman ME Lacroix J Odetola FO. Epidemiology and outcomes of pediatric multiple organ dysfunction syndrome. Pediatr Crit Care Med (2017) 18(3_suppl Suppl 1):S4–S16. doi: 10.1097/PCC.0000000000001047 28248829 PMC5334773

[5] Hall MW Geyer SM Guo CY Panoskaltsis-Mortari A Jouvet P Ferdinands J. Innate immune function and mortality in critically ill children with influenza: a multicenter study. Crit Care Med (2013) 41(1):224–36. doi: 10.1097/CCM.0b013e318267633c PMC3705720 23222256

[6] Novak T Hall MW McDonald DR Newhams MM Mistry AJ Panoskaltsis-Mortari A. RIG-I and TLR4 responses and adverse outcomes in pediatric influenza-related critical illness. J Allergy Clin Immunol (2020) 145(6):1673–1680 e1611. doi: 10.1016/j.jaci.2020.01.040 32035159 PMC7323584

[7] Fiore-Gartland A Panoskaltsis-Mortari A Agan AA Mistry AJ Thomas PG Matthay MA. Cytokine profiles of severe influenza virus-related complications in children. Front Immunol (2017) 8:1423. doi: 10.3389/fimmu.2017.01423 29163498 PMC5681736

[8] Kangelaris KN Prakash A Liu KD Aouizerat B Woodruff PG Erle DJ. Increased expression of neutrophil-related genes in patients with early sepsis-induced ARDS. Am J Physiol Lung Cell Mol Physiol (2015) 308(11):L1102–1113. doi: 10.1152/ajplung.00380.2014 PMC4451399 25795726

[9] Sweeney TE Wong HR Khatri P. Robust classification of bacterial and viral infections via integrated host gene expression diagnostics. Sci Transl Med (2016) 8, 346ra91–346ra91. doi: 10.1126/scitranslmed.aaf7165 PMC5348917 27384347

[10] Wong HR Cvijanovich NZ Anas N Allen GL Thomas NJ Bigham MT. Improved risk stratification in pediatric septic shock using both protein and mRNA biomarkers. PERSEVERE-XP. Am J Respir Crit Care Med (2017) 196(4):494–501. doi: 10.1164/rccm.201701-0066OC 28324661 PMC5564676

[11] Matics TJ Sanchez-Pinto LN. Adaptation and validation of a pediatric sequential organ failure assessment score and evaluation of the sepsis-3 definitions in critically ill children. JAMA Pediatr (2017) 171(10):e172352. doi: 10.1001/jamapediatrics.2017.2352 28783810 PMC6583375

[12] Canouil M Bouland GA Bonnefond A Froguel P t Hart LM Slieker RC. NACHO: an r package for quality control of NanoString nCounter data. Bioinformatics. (2020) 36(3):970–1. doi: 10.1093/bioinformatics/btz647 PMC9883715 31504159

[13] Ritchie ME Phipson B Wu D Hu Y Law CW Shi W. Limma powers differential expression analyses for RNA-sequencing and microarray studies. Nucleic Acids Res (2015) 43(7):e47. doi: 10.1093/nar/gkv007 25605792 PMC4402510

[14] Hahn G Novak T Crawford JC Randolph AG Lange C. Longitudinal analysis of contrasts in gene expression data. Genes. (2023) 14(6):1134. doi: 10.3390/genes14061134 37372314 PMC10298400

[15] Dunning J Blankley S Hoang LT Cox M Graham CM James PL. Progression of whole-blood transcriptional signatures from interferon-induced to neutrophil-associated patterns in severe influenza. Nat Immunol (2018) 19(6):625–35. doi: 10.1038/s41590-018-0111-5 PMC5985949 29777224

[16] Perpinan C Bertran L Terra X Aguilar C Binetti J Lopez-Dupla M. Resistin and IL-15 as predictors of invasive mechanical ventilation in COVID-19 pneumonia irrespective of the presence of obesity and metabolic syndrome. J Pers Med (2022) 12(3):391. doi: 10.3390/jpm12030391 PMC8955294 35330391

[17] Kruzel ML Zimecki M Actor JK. Lactoferrin in a context of inflammation-induced pathology. Front Immunol (2017) 8:1438. doi: 10.3389/fimmu.2017.01438 29163511 PMC5681489

[18] Sweeney TE Thomas NJ Howrylak JA Wong HR Rogers AJ Khatri P. Multicohort analysis of whole-blood gene expression data does not form a robust diagnostic for acute respiratory distress syndrome. Crit Care Med (2018) 46(2):244–51. doi: 10.1097/CCM.0000000000002839 PMC5774019 29337789

[19] Velasquez SY Coulibaly A Sticht C Schulte J Hahn B Sturm T. Key signature genes of early terminal granulocytic differentiation distinguish sepsis from systemic inflammatory response syndrome on intensive care unit admission. Front Immunol (2022) 13:864835. doi: 10.3389/fimmu.2022.864835 35844509 PMC9280679

[20] Sanyal R Polyak MJ Zuccolo J Puri M Deng L Roberts L. MS4A4A: a novel cell surface marker for M2 macrophages and plasma cells. Immunol Cell Biol (2017) 95(7):611–9. doi: 10.1038/icb.2017.18 28303902

[21] Li Y Li Y Bai Z Pan J Wang J Fang F. Identification of potential transcriptomic markers in developing pediatric sepsis: a weighted gene co-expression network analysis and a case-control validation study. J Transl Med (2017) 15(1):254. doi: 10.1186/s12967-017-1364-8 29237456 PMC5729245

[22] Roy JG McElhaney JE Verschoor CP. Reliable reference genes for the quantification of mRNA in human T-cells and PBMCs stimulated with live influenza virus. BMC Immunol (2020) 21(1):4. doi: 10.1186/s12865-020-0334-8 32005148 PMC6995044

[23] Rayes J Watson SP Nieswandt B. Functional significance of the platelet immune receptors GPVI and CLEC-2. J Clin Invest. (2019) 129(1):12–23. doi: 10.1172/JCI122955 30601137 PMC6307936

[24] Sfikakis PP Verrou KM Ampatziadis-Michailidis G Tsitsilonis O Paraskevis D Kastritis E. Blood transcriptomes of anti-SARS-CoV-2 antibody-positive healthy individuals who experienced asymptomatic versus clinical infection. Front Immunol (2021) 12:746203. doi: 10.3389/fimmu.2021.746203 34675930 PMC8523987

[25] Parnell G McLean A Booth D Huang S Nalos M Tang B. Aberrant cell cycle and apoptotic changes characterise severe influenza a infection–a meta-analysis of genomic signatures in circulating leukocytes. PloS One (2011) 6(3):e17186. doi: 10.1371/journal.pone.0017186 21408152 PMC3050844

[26] Monaco G Lee B Xu W Mustafah S Hwang YY Carré C. RNA-Seq signatures normalized by mRNA abundance allow absolute deconvolution of human immune cell types. Cell Rep (2019) 26(6):1627–1640.e1627. doi: 10.1016/j.celrep.2019.01.041 30726743 PMC6367568

[27] Sweeney TE Wynn JL Cernada M Serna E Wong HR Baker HV. Validation of the sepsis MetaScore for diagnosis of neonatal sepsis. J Pediatr Infect Dis Soc (2018) 7(2):129–35. doi: 10.1093/jpids/pix021 PMC5954302 28419265

[28] Markovic SS Gajovic N Jurisevic M Jovanovic M Jovicic BP Arsenijevic N. Galectin-1 as the new player in staging and prognosis of COVID-19. Sci Rep (2022) 12(1):1272. doi: 10.1038/s41598-021-04602-z 35075140 PMC8786829

[29] Yang ML Chen YH Wang SW Huang YJ Leu CH Yeh NC. Galectin-1 binds to influenza virus and ameliorates influenza virus pathogenesis. J Virol (2011) 85(19):10010–20. doi: 10.1128/JVI.00301-11 PMC3196456 21795357

[30] Lang R Raffi FAM. Dual-specificity phosphatases in immunity and infection: an update. Int J Mol Sci (2019) 20(11):2710. doi: 10.3390/ijms20112710 PMC6600418 31159473

[31] Yu M Li G Lee WW Yuan M Cui D Weyand CM. Signal inhibition by the dual-specific phosphatase 4 impairs T cell-dependent b-cell responses with age. Proc Natl Acad Sci U S A. (2012) 109(15):E879–888. doi: 10.1073/pnas.1109797109 PMC3326453 22434910

[32] Sweeney TE Azad TD Donato M Haynes WA Perumal TM Henao R. Unsupervised analysis of transcriptomics in bacterial sepsis across multiple datasets reveals three robust clusters. Crit Care Med (2018) 46(6):915–25. doi: 10.1097/CCM.0000000000003084 PMC5953807 29537985

[33] Zhai Y Franco LM Atmar RL Quarles JM Arden N Bucasas KL. Host transcriptional response to influenza and other acute respiratory viral infections–a prospective cohort study. PloS Pathog (2015) 11(6):e1004869. doi: 10.1371/journal.ppat.1004869 26070066 PMC4466531

[34] Ramilo O Allman W Chung W Mejias A Ardura M Glaser C. Gene expression patterns in blood leukocytes discriminate patients with acute infections. Blood. (2007) 109(5):2066–77. doi: 10.1182/blood-2006-02-002477 PMC1801073 17105821

[35] Galmiche L Serre V Beinat M Assouline Z Lebre AS Chretien D. Exome sequencing identifies MRPL3 mutation in mitochondrial cardiomyopathy. Hum Mutat (2011) 32(11):1225–31. doi: 10.1002/humu.21562 21786366

[36] van der Windt GJ Everts B Chang CH Curtis JD Freitas TC Amiel E. Mitochondrial respiratory capacity is a critical regulator of CD8+ T cell memory development. Immunity. (2012) 36(1):68–78. doi: 10.1016/j.immuni.2011.12.007 22206904 PMC3269311

[37] van der Windt GJ O'Sullivan D Everts B Huang SC Buck MD Curtis JD. CD8 memory T cells have a bioenergetic advantage that underlies their rapid recall ability. Proc Natl Acad Sci U S A. (2013) 110(35):14336–41. doi: 10.1073/pnas.1221740110 PMC3761631 23940348

[38] Chang Y He J Tang J Chen K Wang Z Xia Q. Investigation of the gene co-expression network and hub genes associated with acute mountain sickness. Hereditas. (2020) 157(1):13. doi: 10.1186/s41065-020-00127-z 32299499 PMC7164164

[39] Abbas M AK Lichtman, MD, PhD AH Pillai, MBBS, PhD S. Cellular and molecular immunology, 10th edition. 10th ed. USA: Elsevier, Inc (2022). p. 618.

[40] Liu S Huang Z Deng X Zou X Li H Mu S. Identification of key candidate biomarkers for severe influenza infection by integrated bioinformatical analysis and initial clinical validation. J Cell Mol Med (2021) 25(3):1725–38. doi: 10.1111/jcmm.16275 PMC7875920 33448094

[41] Tang BM Shojaei M Teoh S Meyers A Ho J Ball TB. Neutrophils-related host factors associated with severe disease and fatality in patients with influenza infection. Nat Commun (2019) 10(1):3422. doi: 10.1038/s41467-019-11249-y 31366921 PMC6668409

[42] Zerbib Y Jenkins EK Shojaei M Meyers AFA Ho J Ball TB. Pathway mapping of leukocyte transcriptome in influenza patients reveals distinct pathogenic mechanisms associated with progression to severe infection. BMC Med Genomics (2020) 13(1):28. doi: 10.1186/s12920-020-0672-7 32066441 PMC7027223

[43] He JQ Sandford AJ Wang IM Stepaniants S Knight DA Kicic A. Selection of housekeeping genes for real-time PCR in atopic human bronchial epithelial cells. Eur Respir J (2008) 32(3):755–62. doi: 10.1183/09031936.00129107 18417509

[44] Alexandropoulou O Kossiva L Haliotis F Giannaki M Tsolia M Panagiotou IP. Transient neutropenia in children with febrile illness and associated infectious agents: 2 years' follow-up. Eur J Pediatr (2013) 172(6):811–9. doi: 10.1007/s00431-013-1965-z 23408310

[45] Brown KA Brain SD Pearson JD Edgeworth JD Lewis SM Treacher DF. Neutrophils in development of multiple organ failure in sepsis. Lancet. (2006) 368(9530):157–69. doi: 10.1016/S0140-6736(06)69005-3 16829300

[46] Kumar S Payal N Srivastava VK Kaushik S Saxena J Jyoti A. Neutrophil extracellular traps and organ dysfunction in sepsis. Clin Chim Acta (2021) 523:152–62. doi: 10.1016/j.cca.2021.09.012 34537216

[47] Brass AL Huang IC Benita Y John SP Krishnan MN Feeley EM. The IFITM proteins mediate cellular resistance to influenza a H1N1 virus, West Nile virus, and dengue virus. Cell. (2009) 139(7):1243–54. doi: 10.1016/j.cell.2009.12.017 PMC2824905 20064371

