# Supplementary material for: Transcriptomic profiles of multiple organ dysfunction syndrome phenotypes in pediatric critical influenza
Source: Front Immunol. 2023 Jul 18;14:1220028. doi: 10.3389/fimmu.2023.1220028 (PMC10390830; doi:10.3389/fimmu.2023.1220028)
Supplement: Supplementary file 1 [file DataSheet_1.pdf]

Supplementary Material

Title: Transcriptomic Profiles of Multiple Organ Dysfunction Syndrome Phenotypes in Pediatric Critical Influenza

Tanya Novak[1,2,3], Jeremy Chase Crawford[1,4,5], Georg Hahn[6], Mark W. Hall[7], Simone A. Thair[1,8], Margaret M. Newhams[1,3] Janet Chou[9,10], Peter M. Mourani[11], Keiko M. Tarquinio[12], Barry Markovitz[13], Laura L. Loftis[14], Scott L. Weiss[15], Renee Higgerson[16], Adam J. Schwarz[17], Neethi P. Pinto[18], Neal J. Thomas[19], Rainer G. Gedeit[20], Ronald C. Sanders, Jr. [21], Sidharth Mahapatra[22], Bria M. Coates[23], Natalie Z. Cvijanovich[24], Kate G. Ackerman[25], David Tellez[26], Patrick McQuillen[27], Stephen C. Kurachek[28], Steven L. Shein[29], Christoph Lange[30], Paul G. Thomas[3,5] and Adrienne G. Randolph[1,2,3,10]* on behalf of the Pediatric Acute Lung Injury Sepsis Investigators (PALISI) Network and the Pediatric Intensive Care Influenza (PICFLU) Investigators

[1]Department of Anesthesiology, Critical Care and Pain Medicine, Boston Children's Hospital, Boston, MA, United States, [2]Department of Anaesthesia, Harvard Medical School, Boston, MA, United States. [3]NIAID, Centers of Excellence for Influenza Research and Response (CEIRR), Center for Influenza Disease and Emergence Response (CIDER), Athens, GA, United States, [4]NIAID, Centers of Excellence for Influenza Research and Response (CEIRR), St. Jude Children's Research Hospital, Memphis, TN, United States. [5]Department of Immunology, St Jude Children's Research Hospital, Memphis, TN, United States, [6]Channing Division of Network Medicine, Department of Medicine, Brigham and Women's Hospital, Boston, MA, [7]Division of Critical Care Medicine, Department of Pediatrics, Nationwide Children's Hospital, Columbus, OH, [8]Division of Biomedical Informatics Research, Department of Medicine, Stanford University School of Medicine, Stanford, CA, [9]Division of Immunology, Boston Children's Hospital, Harvard Medical School, Boston, MA, United States, [10]Department of Pediatrics, Harvard Medical School, Boston, MA, United States, [11]Department of Pediatrics, Section of Critical Care Medicine, University of Arkansas for Medical Sciences and Arkansas Children's Research Institute, Little Rock, AR, United States, [12]Division of Critical Care Medicine, Department of Pediatrics, Emory University School of Medicine, Children's Healthcare of Atlanta, Atlanta, GA, United States, [13]Department of Anesthesiology Critical Care Medicine, Children's Hospital Los Angeles, Los Angeles, CA, United States, [14]Division of Critical Care Medicine, Department of Pediatrics, Baylor College of Medicine, Houston, TX, United States, [15]Nemours Children's Hospital Delaware, Critical Care Medicine, Wilmington, DE, United States, [16]Pediatric Critical Care Medicine, St. David's Children's Hospital, Austin, TX, United States, [17]Department of Pediatrics, Children's Hospital of Orange County, Orange, CA, United States, [18]Department of Anesthesiology and Critical Care Medicine, Children's Hospital of Philadelphia, Philadelphia, PA,

United States, [19]Department of Pediatrics, Penn State Health Children's Hospital, Penn State University College of Medicine, Hershey, PA, United States, [20]Pediatric Critical Care, Milwaukee Hospital-Children's Wisconsin, Milwaukee, WI, [21]Section of Pediatric Critical Care, Department of Pediatrics, University of Arkansas for Medical Sciences and Arkansas Children's Research Institute, Little Rock, AR, United States, [22]Pediatric Critical Care Medicine, Children's Hospital & Medical Center Omaha, University of Nebraska Medical Center, Omaha, NE, United States, [23]Division of Critical Care Medicine, Department of Pediatrics, Northwestern University Feinberg, School of Medicine, Ann & Robert H. Lurie Children's Hospital of Chicago, Chicago, IL, United States, [24]Division of Critical Care Medicine, UCSF Benioff Children's Hospital, Oakland, CA, United States, [25]Department of Pediatrics, University of Rochester/UR Medicine Golisano Children's Hospital, Rochester, NY, United States, [26]Pediatric Critical Care Medicine, Phoenix Children's Hospital, Phoenix, AZ, United States, [27]Department of Pediatrics, Benioff Children's Hospital, University of California, San Francisco, San Francisco, CA, United States, [28]Department of Critical Care, Children's Specialty Center, Children's Minnesota, Minneapolis, MN, United States, [29]Division of Pediatric Critical Care Medicine, University Hospitals Rainbow Babies and Children's Hospital, Cleveland, OH, United States, [30]Department of Biostatistics, T.H. Chan School of Public Health, Harvard University, Boston, MA, United States, on behalf of the Pediatric Acute Lung Injury and Sepsis Investigators (PALISI) Network and the Pediatric Intensive Care Influenza (PICFLU) Investigators[§]

*** Correspondence:** Corresponding Author: Dr Adrienne G. Randolph, MS, MD

Adrienne.Randolph@childrens.harvard.edu

METHODS

Patients from the original PICFLU Study (1) were selected to be included in this study using the following inclusion/exclusion criteria:

I. Patient Inclusion Criteria

1. <18 years old
2. Admission to an ICU
3. Symptomatic for acute severe viral infection by **at least one** of the following:

    a. Lower respiratory tract infection (LRTI) with any of the following: hypoxia, hypercarbia, infiltrates on chest radiograph, respiratory failure, respiratory insufficiency or severe distress, tachypnea, or retractions.

    b. Shock treated with vasoactive agents (dopamine, dobutamine, epinephrine, norepinephrine, phenylephrine) and receiving antibiotics due to clinical suspicion of infection

    c. Central nervous system dysfunction (altered mental status or clinical suspicion of meningitis, encephalitis, or encephalopathy) plus fever (temp ≥ 38° C) and cough or sore throat

    d. Acute increase in respiratory support, including any of the following:

        i. Continuous intravenous (IV) or inhaled beta-agonist therapy (albuterol, terbutaline, etc.) for severe bronchospasm

ii. Mechanical ventilator support via a mask or endotracheal tube or tracheostomy tube

iii. High-flow nasal cannula oxygen support

4. Influenza virus confirmed by RT-PCR, GenMark Diagnostics Inc Multiple Respiratory Viral Panel (Carlsbad, CA), multiplex PCR-based BioFire FilmArray® (Salt Lake City, UT), point of care rapid tests or direct fluorescent antigen (the latter two did not provide subtypes).

5. Clinical data was available for pSOFA scoring

6. Parent or legal guardian able and willing to provide permission.

II. Patient Exclusion Criteria

1. Inability to collect respiratory specimen for RT-PCR testing within 7 days of illness onset.
2. Nosocomial-acquired infection as determined at the study site by infection control group.
3. Neuromuscular disease requiring chronic mechanical ventilator support through a mask or tracheostomy for neuromuscular weakness.
4. Chronic mechanical ventilator support through a tracheostomy for chronic respiratory failure.
5. End-stage lung disease being evaluated or awaiting lung transplant.
6. Evidence of current pregnancy from clinical management or other documentation.
7. Any of the following pre-existing conditions: immunodeficiencies, chronic lung disease, symptomatic cardiac disease, neuromuscular disease, malignancy, metabolic or mitochondrial disease, or patients who received systemic immunosuppressive medications within the six weeks prior to admission for this acute illness.

pSOFA Scoring

For the Pediatric Sequential Organ Failure Assessment (pSOFA) respiratory scoring in this study, patients on extracorporeal membrane oxygenation (ECMO) support were assigned the maximum respiratory score of 4. Patients with a PaO_2/FiO_2 (P/F ratio) less than 200 without respiratory support were given a score of respiratory score 2. For patients not receiving vasopressor support (e.g., mean arterial pressure was not available), systolic blood pressure was used to assign a cardiovascular score of 0 or 1 by applying the 2005 international pediatric sepsis and organ dysfunction guidelines (2). A score of 2 or greater qualified as organ dysfunction for the pSOFA score (range 0-4 per organ system).

Multi-Cohort Meta-Analysis Discovery

To expand on the previously published Influenza MetaSignature (IMS) (3), we performed a systematic search for publicly available human gene expression data in peripheral blood and incorporated newer influenza studies. Using the metaIntegrator package in R (v. 2.0.0) (4, 5) in 7 datasets containing 623 samples, we performed multicohort meta-analysis as described previously (3, 6-8) resulting in a signature of 152 genes (unpublished data). These are listed as "Influenza-associated genes" under Gene Source column in **Table S1**.

RNA Extraction

For RNA extraction, the PAXgene Blood RNA Kit (Qiagen, Waltham, MA) was used with the QIAcube instrument as per manufacturer's guidelines with minor modifications based on blood volume and white blood count (WBC). If WBC count ($x10^6$ WBC/ml) was obtained close to the blood collection time, it was multiplied by the volume of blood (ml) to estimate the total number of cells. If the estimated total WBC count was between $12x10^6$ - $27.5x10^6$, then the original

protocol was followed as per instructions with 80µl final elution. If the total WBC count was <12x10^6, then BR5 buffer was omitted from QIAcube setup; instead, after the run was complete, 40 µl of BR5 buffer was added manually to the spin column and centrifuged for 1 min at top speed to elute RNA. The RNA sample was then placed back into the QIAcube to incubate at 65°C for 5 min as per instructions. If the WBC count was ≥27.5x10^6, the sample was split evenly into two tubes after the second centrifugation step by resuspending the pellet with 700 µl of BR1 (instead of 350 µl). The QIAcube was set up to use two separate spin columns. If no WBC count was available, the resuspended cell pellet was assessed for opacity (increased opacity = increased cellular material) to determine whether to split the sample or follow the original protocol.

All RNA extracts were tested for quality and quantity using the Agilent 2100 Bioanalyzer and Agilent RNA 6000 Nano Kit (Agilent Technologies, Inc., Santa Clara, CA) per the manufacturer's instructions. RNA samples were then aliquoted and stored at -80°C until use. The PAXgene Blood RNA Kit incorporated an on-column DNase digestion, with additional DNase treatment applied as needed following quality assessment using the DNA-free™ kit (Ambion, Life Technologies, Carlsbad, CA). RNA analysis was again validated on the Bioanalyzer before use.

NanoString

Reporter and Capture ProbeSets were manufactured within the same lot to reduce variability. Incorporated into these reagents were the NanoString manufactured 6 positive controls and 8 negative controls so that each sample reported control values. To prepare the RNA for the NanoString run, each sample was diluted to 17 ng/µl in RNase-free water (Qiagen) and 5 µl added into small PCR tubes with 8 µl of a mastermix (containing 3 ul Reporter CodeSet and 5 µl hybridization buffer) followed by 2 µl of Capture ProbeSet. After capping the tubes to mix by inversion, they were pulse centrifuged and incubated for16 hours overnight at 65°C (70°C heated

lid) on a thermal cycler (details) then stabilized at 4°C and run within 24 hours. To obtain a loading volume of 30 µl, 15 µl of RNase-free water was added to each sample.

Initial Gene Expression Analysis and Quality Control

The NanoString custom panel included a total of 500 mRNA targets: 469 designed for human mRNA detection and 31 for viral/bacterial detection. The latter were included in the normalization but not reported for analysis as they were outside the scope of this study. During quality control, the housekeeping gene, *FPGS*, was excluded due to low expression and high coefficient of variation. *GUSB* was also excluded due to its expression being statistically increased in the Prolonged MODS patient group. Samples were excluded from analysis if they produced values outside of the accepted quality control range for NanoString defined technical cartridge characteristics including binding density, field of view, negative and positive control marker counts, or positive normalization factor, and/or if they were below the limit of detection. A final 214 influenza positive patients with a single, early sample were included in the normalization.

Statistical analysis for paired longitudinal samples

A method was developed to analyze the paired longitudinal data between two groups by Hahn, et al (9). The 469 genes were assessed for 45 longitudinal paired samples using linear regression with an intercept of the MODS outcome on the normalized mRNA counts per gene for all subjects, where log10 age was included as a single covariate. Contrasts (defined as the difference in measurements between two timepoints) were computed for all subjects falling into the categories "MODS recovery" (group 1) and "Prolonged MODS" (group 2). The null distribution for the intercept of the contrast in the linear model is a t-distribution with n-2 degrees of freedom, where n is the number

of subjects to be tested (that is, the size of either group 1 or group 2). We aimed to test the intersection hypothesis consisting of "MODS recovery” and the complement of “Prolonged MODS". To this end, we define two hypotheses. First, for recovered MODS (group 1) we tested the null hypothesis that the intercept is zero against the complementary alternative that the intercept is non-zero. For “Prolonged MODS” (group 2), we tested the alternative hypothesis that the intercept is larger (more extreme) than some level L. For this, we fit a linear regression with intercept to the contrasts with $\log_{10}$ age as covariate and recorded the R^2 of the resulting model. We then chose L by scaling the intercept for group 2 until we explained 25% of the initial R^2.

Both the tests for group 1 (under the null) and group 2 (under alternative) yielded one p-value per gene. We evaluate all 469 p-values per group separately. That is, we evaluated the 469 p-values in group 1 using the Benjamini-Hochberg procedure to correct for multiple testing using the FDR criterion (10). Likewise, we applied Benjamini-Hochberg to the 469 p-values for group 2. Each time, the Benjamini-Hochberg procedure was applied using a threshold of 0.05/2. We report those genes that are significant in both groups after the multiple testing correction.

RESULTS

Upregulation of *LGALS1, GNA15, DUSP4,* and *TWISTNB* in Prolonged MODS

Although *LGALS1*, *GNA15*, *DUSP4*, and *TWISTNB* were upregulated in Prolonged MODS patients, their significance was the least robust and therefore not as definitive as the other genes. Still, they were able to differentiate the patients that recovered from MODS within 7 days as opposed to those that had extended organ dysfunction. *LGALS1* and *GNA15* are known as for their roles in immune regulation and homeostasis. The protein Galectin-1 produced by *LGALS1* has been shown to bind various influenza A subtypes *in vitro* and improve survival in infected mice (11) and

was suggested as a serum cytokine marker for severe COVID-19 (12). Upregulated *GNA15* has been proven to be prognostic (along with 10 other biomarkers) for sepsis in neonates (13). *DUSP4* is an immune regulator highly expressed by T regs and also controls T helper cell proliferation and differentiation (14). Overexpression of *DUSP4* mRNA has been shown to cause defective signaling of T cell-dependent B-cell responses such as decreased antibody production following influenza vaccination in the elderly (15). In the context of sepsis, DUSP4-deficient mice show improved survival (14). Therefore, the increase of *DUSP4* in our Prolonged MODS group in which 24% of the children died reflects its detriment in this cohort. Lastly, upregulation of *TWISTNB,* which encodes a component of RNA polymerase I that helps catalyze the transcription of DNA into RNA, was expected to reflect lower clinical severity and decreased mortality rate as described in Sweeney, *et al*'s, analysis on bacterial sepsis (16) however this was not the case in our cohort. As a fundamental cellular process, its upregulation reflects increased leukocyte ribosomal RNA production which ultimately helped to differentiate the Prolonged MODS group.

Supplementary Table S2. Number of patients who tested positive for a bacterial co-infection that had Prolonged MODS (n=26/38), recovered from MODS within 7 days (n=8/27), and those that never developed MODS (n=28/126).

MODS Group	Culture source	Positive Results*	Organisms
**Prolonged MODS**	Blood	1	1 MRSA
	LRT	22	13 MRSA<br>8 MSSA (incl 2 dual infections with Streptococcus pneumoniae)<br>1 Group A Streptococcus
	Nasopharyngeal	2	1 MRSA<br>1 Group A Streptococcus
	Pleural Fluid	1	1 Group A Streptococcus
**Recovered MODS**	Blood	1	1 Streptococcus pneumoniae
	LRT	7	1 MRSA<br>5 MSSA (incl 2 dual infections with Streptococcus pneumoniae)<br>1 Group A Streptococcus
**Never MODS**	Blood	3	1 MRSA<br>1 Streptococcus pneumoniae + Moraxella catarrhalis<br>1 Mycoplasma pneumoniae
	LRT	20	2 MRSA + 1 Streptococcus pneumoniae<br>11 MSSA + 1 Streptococcus pneumoniae<br>3 Haemophilus influenzae non-typeable<br>1 Haemophilus influenzae type b<br>1 Streptococcus pneumoniae + 1 Moraxella catarrhalis<br>1 Mycoplasma pneumoniae<br>1 Gram negative rods and Gram positive cocci
	Nasopharyngeal	3	1 MSSA<br>1 Streptococcus pneumoniae<br>1 Group A Streptococcus
	Wound/Other	2	1 MRSA<br>1 Group A Streptococcus

*Considered a true infection based on bacterial culture results. **MODS**, Multiple organ dysfunction syndrome; **LRT**, Lower respiratory tract; **MRSA**, Methicillin-resistant Staphylococcus aureus; **MSSA**, Methicillin-sensitive Staphylococcus aureus.

Supplementary Table S3A. Prolonged/Died MODS patients had 94 mRNA levels that were significantly higher compared to Never MODS.

Gene	Log2 Fold Change	P value	FDR adjusted P value	Significant differences
**RETN**	958.5845	3.45E-16	1.72E-13	****
**GAPDH**	5355.489	3.82E-15	9.56E-13	****
**TCN1**	699.6071	4.61E-13	7.68E-11	****
**MS4A4A**	832.8943	7.96E-13	9.95E-11	****
**LCN2**	2530.722	2.48E-12	2.48E-10	****
**BPI**	473.6566	7.39E-12	6.16E-10	****
**MMP8**	3396.607	1.25E-11	8.91E-10	****
**OLFM4**	1080.422	1.80E-11	1.12E-09	****
**ESPL1**	23.01569	7.49E-10	4.16E-08	****
**ZDHHC19**	441.7068	5.17E-09	2.59E-07	****
**GUSB**	86.64937	6.24E-09	2.79E-07	****
**LTF**	671.476	6.71E-09	2.79E-07	****
**S100A12**	50163.32	7.80E-09	3.00E-07	****
**LGALS1**	1620.841	9.93E-09	3.55E-07	****
**MMP9**	1610.877	1.58E-08	5.25E-07	****
**CLEC1B**	34.51097	3.00E-08	9.38E-07	****
**DUSP4**	33.12819	3.30E-08	9.72E-07	****
**GNA15**	25.91465	4.39E-08	1.22E-06	****
**BATF**	61.1541	1.21E-07	3.18E-06	****
**TIMP1**	638.6034	2.32E-07	5.81E-06	****
**TGFBI**	-139.039	3.01E-07	7.17E-06	****
**EMP1**	28.20806	4.13E-07	9.39E-06	****
**S100A9**	138544.2	6.34E-07	1.38E-05	****
**LY86**	-135.325	8.02E-07	1.67E-05	****
**IL1A**	19.60507	8.95E-07	1.77E-05	****
**ADORA2A**	67.6408	9.22E-07	1.77E-05	****
**CD177**	3652.952	1.12E-06	2.07E-05	****
**IL13**	12.44043	1.34E-06	2.39E-05	****
**SLC12A7**	-20.7055	4.20E-06	7.09E-05	****
**HLA-DMB**	-167.675	4.25E-06	7.09E-05	****
**HPRT1**	48.68952	4.47E-06	7.22E-05	****
**TWISTNB**	39.094	6.69E-06	0.0001	****
**HK3**	756.4483	7.48E-06	0.0001	****
**H1F0**	113.2142	1.14E-05	0.0002	***
**S100A8**	61196.77	1.17E-05	0.0002	***
**YKT6**	26.98738	1.23E-05	0.0002	***

**CTSL1**	45.76675	2.38E-05	0.0003	***
**RBP7**	-80.4159	4.62E-05	0.0006	***
**STING**	98.0413	5.70E-05	0.0007	***
**TNFA**	46.08042	6.18E-05	0.0008	***
**FURIN**	48.04847	6.75E-05	0.0008	***
**PLEKHO1**	-144.871	8.30E-05	0.001	***
**ELANE**	62.3986	0.000114	0.001	***
**MPO**	36.02736	0.000119	0.001	***
**ANXA1**	1719.572	0.000154	0.002	**
**IL15RA**	48.2579	0.000157	0.002	**
**IL16**	-275.473	0.000168	0.002	**
**IL10**	15.16616	0.000169	0.002	**
**HAL**	-187.974	0.000186	0.002	**
**STX1A**	6.634984	0.000211	0.002	**
**TULP2**	6.303997	0.000275	0.003	**
**DR1**	38.95784	0.000279	0.003	**
**NUDT1**	30.47757	0.000307	0.003	**
**HLADRA**	-678.015	0.000314	0.003	**
**ZC3H12A**	44.96297	0.000332	0.003	**
**BTN2A2**	-14.9221	0.000351	0.003	**
**STAB1**	-8.92136	0.000431	0.004	**
**DEFA1/1B/3**	17502.98	0.000432	0.004	**
**HIST1H2AM**	680.2377	0.000645	0.01	**
**DEFB114**	4.824773	0.000829	0.01	**
**NFKB1**	38.23309	0.000951	0.01	**
**HIST2H2BE**	-163.945	0.001038	0.01	**
**CD24**	674.4025	0.001196	0.01	**
**ATOX1**	26.65179	0.001269	0.01	**
**EBI3**	5.63417	0.001376	0.01	**
**HLA-E**	-1724.72	0.001448	0.01	**
**CASP1**	-405.614	0.001671	0.01	**
**CEACAM1**	385.6801	0.001687	0.01	**
**SAP30**	-215.622	0.001774	0.01	**
**IL17A**	1.375368	0.002019	0.01	**
**CCL5**	-188.566	0.002161	0.01	**
**P2RX1**	19.55354	0.00231	0.02	*
**C3AR1**	197.3992	0.002401	0.02	*
**TP53BP1**	-13.4984	0.00249	0.02	*
**GADD45A**	255.6322	0.002503	0.02	*
**DEFA4**	554.8239	0.003043	0.02	*
**CAT**	277.7465	0.003213	0.02	*

**EVI2A**	-363.323	0.003804	0.02	*
**IRF5**	-26.5845	0.005135	0.03	*
**PRKCDBP**	5.971611	0.005217	0.03	*
**CRISP2**	9.201002	0.00546	0.03	*
**KCNJ2**	-164.027	0.005989	0.03	*
**FXYD6**	-11.9194	0.006897	0.04	*
**CASP5**	-166.169	0.0073	0.04	*
**IFIT5**	-477.879	0.007783	0.04	*
**TLR6**	-129.319	0.008121	0.05	*
**TLR9**	-9.52089	0.008411	0.05	*
**CSF3**	4.440344	0.00865	0.05	*
**APOE**	0.247562	0.008662	0.05	*
**GLRX2**	12.81574	0.008742	0.05	*
**ALDOC**	9.244195	0.008978	0.05	*
**IL9**	1.93305	0.009263	0.05	*
**CASP12**	4.147802	0.009585	0.05	*
**SIRT5**	32.33635	0.010681	0.05	*

****q<0.0001, ***q≤0.001, **q≤0.01, *q<0.05

Table S3B. Nine transcripts with the most similar mRNA expression levels between Prolonged MODS/Died vs Never MODS.

Gene*	Log2 Fold Change	P-value	FDR adjusted P value
**PANX1**	0.293602	0.951807	0.981244045
**IFITM1**	-139.436	0.954355	0.981846691
**RAP2C**	1.168989	0.965603	0.986861066
**GAS6**	0.077949	0.980469	0.995026225
**CD3D**	1.121862	0.980872	0.995026225
**TLR3**	-0.06255	0.983957	0.995908073
**IRS1**	0.013004	0.995623	0.999131042
**B2M**	-130.707	0.995862	0.999131042
**ZCCHC4**	0.017457	0.997908	0.999131042

***Genes included only if they had an average expression of at least 10 mRNA counts or higher after normalization.

Figure S1.

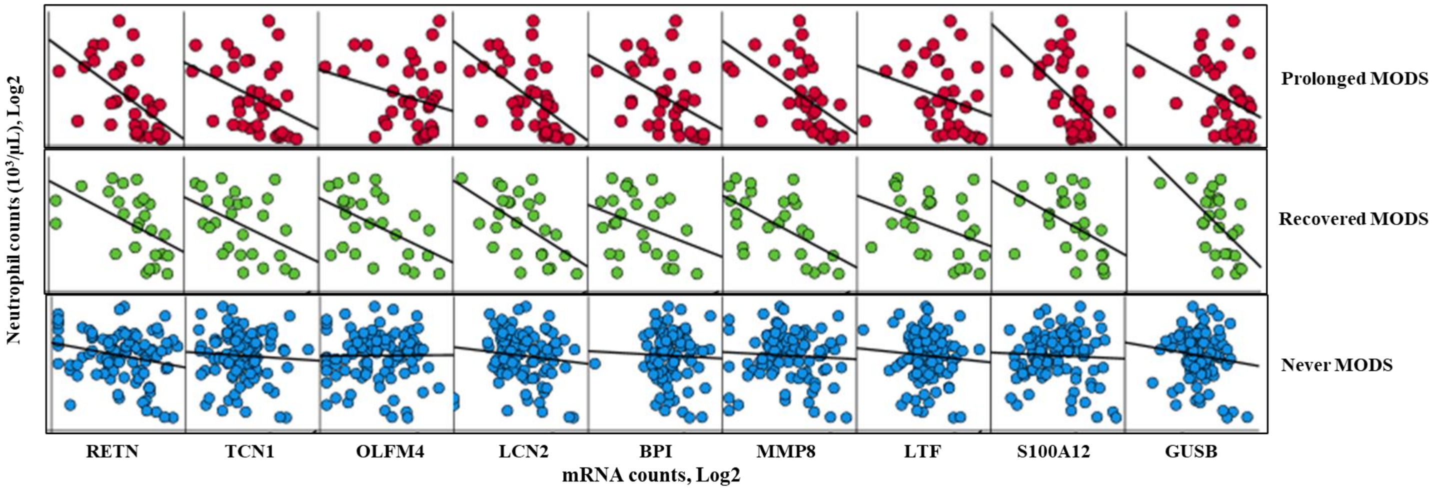

Patient Group	RETN	TCN1	OLFM4	LCN2	BPI	MMP8	LTF	S100A12	GUSB	Spearman's rho
**Prolonged MODS**	-0.657	-0.446	-0.165	-0.643	-0.518	-0.689	-0.4	-0.574	-0.412	Correlation Coefficient
	**<.001**	**0.006**	0.335	**<.001**	**0.001**	**<.001**	**0.016**	**<.001**	**0.013**	Sig. (2-tailed)
**Recovered MODS**	-0.676	-0.469	-0.485	-0.569	-0.329	-0.621	-0.398	-0.543	-0.531	Correlation Coefficient
	**<.001**	**0.016**	**0.012**	**0.002**	0.101	**<.001**	**0.044**	**0.004**	**0.005**	Sig. (2-tailed)
**Never MODS**	-0.186	-0.047	0.043	-0.139	0.027	-0.053	-0.035	0.064	-0.051	Correlation Coefficient
	0.067	0.651	0.677	0.175	0.796	0.606	0.734	0.534	0.618	Sig. (2-tailed)

Figure S1. Correlation matrix of absolute neutrophil counts versus normalized mRNA counts of neutrophil associated degranulation transcripts. Both axes are displayed in Log2. There were missing neutrophil counts for 2/38 Prolonged, 1/27 Recovered, and 29/126 Never MODS patients. Non-parametric Spearman's rho correlation was determined for each MODS group. Significant inverse relationships were evident for all Prolonged MODS and Recovered MODS mRNA transcripts (highlighted in bold in the table) except for *OLFM4* and *BPI*, respectively. The correlation dictates that as neutrophils decreased, neutrophil degranulation mRNA transcripts increased in Prolonged MODS and Recovered MODS patients. Never MODS patients did not have any significant correlations with neutrophil count.

Figure S2.

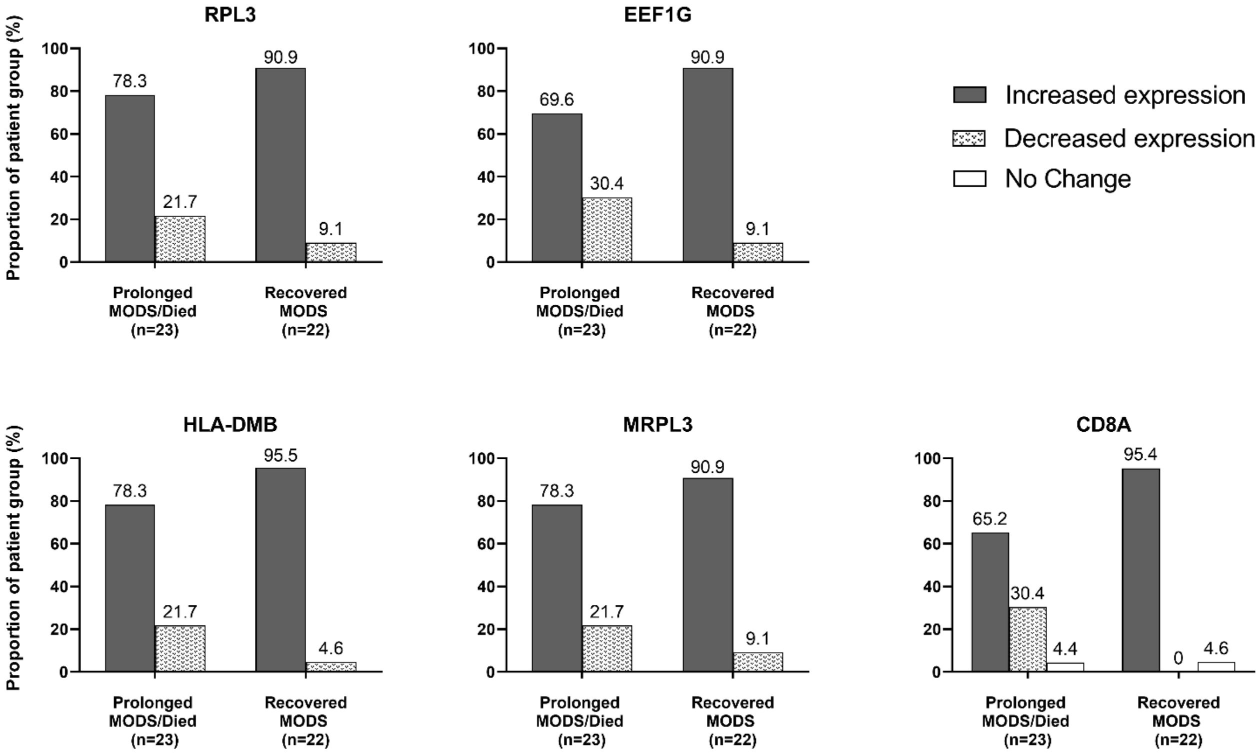

Figure S2. Proportion of Prolonged MODS/Died or Recovered MODS patients with five differentially expressed genes at the second time point. Dark gray bars show the percentage of patients that had increased mRNA expression of the gene indicated when comparing the first blood collection with the second time point. The light patterned bars show the percentage of patients with decreased mRNA expression. White bars indicate the percentage of patients with no change in expression which is only evident in CD8A. **RPL3**, Ribosomal protein L3. **EEF1G**, Eukaryotic Translation Elongation Factor 1 Gamma protein. **HLA-DMB**, Major histocompatibility complex, class II, DM beta. **MRPL3**, Mitochondrial Ribosomal Protein L3. **CD8A**, T-cell surface glycoprotein CD8 alpha chain.

References

1. Randolph AG, Agan AA, Flanagan RF, Meece JK, Fitzgerald JC, Loftis LL, *et al.* Optimizing Virus Identification in Critically Ill Children Suspected of Having an Acute Severe Viral Infection. *Pediatr Crit Care Med*. 2016;17(4):279-286.doi:10.1097/PCC.0000000000000661.

2. Goldstein B, Giroir B, Randolph A, International Consensus Conference on Pediatric S. International pediatric sepsis consensus conference: definitions for sepsis and organ dysfunction in pediatrics. *Pediatr Crit Care Med*. 2005;6(1):2-8.doi:10.1097/01.PCC.0000149131.72248.E6.

3. Andres-Terre M, McGuire HM, Pouliot Y, Bongen E, Sweeney TE, Tato CM, *et al.* Integrated, Multi-cohort Analysis Identifies Conserved Transcriptional Signatures across Multiple Respiratory Viruses. *Immunity*. 2015;43(6):1199-1211.doi:10.1016/j.immuni.2015.11.003.

4. Sweeney TE, Haynes WA, Vallania F, Ioannidis JP, Khatri P. Methods to increase reproducibility in differential gene expression via meta-analysis. *Nucleic Acids Res*. 2017;45(1):e1.doi:10.1093/nar/gkw797.

5. Haynes WA, Vallania F, Liu C, Bongen E, Tomczak A, Andres-Terre M, *et al.* Empowering Multi-Cohort Gene Expression Analysis to Increase Reproducibility. *Pac Symp Biocomput*. 2017;22:144-153.doi:10.1142/9789813207813_0015.

6. Khatri P, Roedder S, Kimura N, De Vusser K, Morgan AA, Gong Y, *et al.* A common rejection module (CRM) for acute rejection across multiple organs identifies novel therapeutics for organ transplantation. *J Exp Med*. 2013;210(11):2205-2221.doi:10.1084/jem.20122709.

7. Sweeney TE, Braviak L, Tato CM, Khatri P. Genome-wide expression for diagnosis of pulmonary tuberculosis: a multicohort analysis. *Lancet Respir Med*. 2016;4(3):213-224.doi:10.1016/S2213-2600(16)00048-5.

8. Sweeney TE, Shidham A, Wong HR, Khatri P. A comprehensive time-course-based multicohort analysis of sepsis and sterile inflammation reveals a robust diagnostic gene set. *Sci Transl Med*. 2015;7(287):287ra271.doi:10.1126/scitranslmed.aaa5993.

9. Hahn G, Novak T, Crawford JC, Randolph AG, Lange C. Longitudinal Analysis of Contrasts in Gene Expression Data. *Genes*. 2023;14(6):1134.doi.

10. Benjamini Y, Hochberg Y. Controlling the False Discovery Rate: A Practical and Powerful Approach to Multiple Testing. *Journal of the Royal Statistical Society: Series B (Methodological)*. 1995;57(1):289-300.doi:https://doi.org/10.1111/j.2517-6161.1995.tb02031.x.

11. Yang ML, Chen YH, Wang SW, Huang YJ, Leu CH, Yeh NC, *et al.* Galectin-1 binds to influenza virus and ameliorates influenza virus pathogenesis. *J Virol*. 2011;85(19):10010-10020.doi:10.1128/JVI.00301-11.

12. Markovic SS, Gajovic N, Jurisevic M, Jovanovic M, Jovicic BP, Arsenijevic N, *et al.* Galectin-1 as the new player in staging and prognosis of COVID-19. *Sci Rep*. 2022;12(1):1272.doi:10.1038/s41598-021-04602-z.

13. Sweeney TE, Wynn JL, Cernada M, Serna E, Wong HR, Baker HV, *et al.* Validation of the Sepsis MetaScore for Diagnosis of Neonatal Sepsis. *J Pediatric Infect Dis Soc*. 2018;7(2):129-135.doi:10.1093/jpids/pix021.

14. Lang R, Raffi FAM. Dual-Specificity Phosphatases in Immunity and Infection: An Update. *Int J Mol Sci*. 2019;20(11).doi:10.3390/ijms20112710.

15. Yu M, Li G, Lee WW, Yuan M, Cui D, Weyand CM, *et al.* Signal inhibition by the dual-specific phosphatase 4 impairs T cell-dependent B-cell responses with age. *Proc Natl Acad Sci U S A*. 2012;109(15):E879-888.doi:10.1073/pnas.1109797109.

16. Sweeney TE, Azad TD, Donato M, Haynes WA, Perumal TM, Henao R, *et al.* Unsupervised Analysis of Transcriptomics in Bacterial Sepsis Across Multiple Datasets Reveals Three Robust Clusters. *Crit Care Med*. 2018;46(6):915-925.doi:10.1097/CCM.0000000000003084.
